# Revealing the Diversity of Sequences, Structures, and Targets of Peptides from South China Sea *Macrodactyla doreensis* Based on Transcriptomics

**DOI:** 10.3390/md22100470

**Published:** 2024-10-12

**Authors:** Ziqiang Hua, Yanling Liao, Jinxing Fu, Xinru Li, Qianxia Xu, Limin Lin, Meiling Huang, Bingmiao Gao

**Affiliations:** Engineering Research Center of Tropical Medicine Innovation and Transformation of Ministry of Education, Hainan Key Laboratory for Research and Development of Tropical Herbs, International Joint Research Center of Human-Machine Intelligent Collaborative for Tumor Precision Diagnosis and Treatment of Hainan Province, School of Pharmacy, Hainan Medical University, Haikou 571199, China; huazzq@icloud.com (Z.H.); liaoyanling@hainmc.edu.cn (Y.L.); fjx1900201062@163.com (J.F.); 13588261944@163.com (X.L.); 17757940986@163.com (Q.X.); liminlin239@gmail.com (L.L.)

**Keywords:** *Macrodactyla doreensis*, sea anemone toxins, transcriptomics, AlphaFold2 modeling, molecular docking

## Abstract

The South China Sea is rich in sea anemone resources, and the protein and peptide components from sea anemone toxins comprise an important treasure trove for researchers to search for leading compounds. This study conducted a comprehensive transcriptomic analysis of the tentacles and column of *Macrodactyla doreensis* and explored the distribution and diversity of proteins and peptides in depth using bioinformatics, initially constructing a putative protein and peptide database. In this database, typical peptide families are identified through amino acid sequence analysis, and their 3D structures and potential biological activities are revealed through AlphaFold2 modeling and molecular docking. A total of 4239 transcripts were identified, of which the putative protein accounted for 81.53%. The highest content comprised immunoglobulin and a variety of proteases, mainly distributed in the column and related to biological functions. Importantly, the putative peptide accounted for 18.47%, containing ShK domain and Kunitz-type peptides, mainly distributed in the tentacles and related to offensive predatory behavior. Interestingly, 40 putative peptides belonging to eight typical peptide families were identified, and their structures and targets were predicted. This study reveals the diversity and complexity of *Macrodactyla doreensis* toxins and predicts their structure and targets based on amino acid sequences, providing a feasible approach for research regarding the discovery of peptides with potentially high activity.

## 1. Introduction

Sea anemones are ancient sessile invertebrates, named for their resemblance to sunflowers. They inhabit shallow coral reefs across various marine regions, including the Indian Ocean, the Pacific Ocean, the Red Sea, and the Samoan Islands. Among these regions, the South China Sea, part of the Pacific Ocean, is noted for its particularly high abundance of sea anemones. Sea anemones have traversed an evolutionary path spanning approximately 700 million years, evolving sophisticated sea anemone toxins [1]. Sea anemone toxins are a complex mixture of peptides, proteins, purines, biogenic amines, and assorted compounds, with cysteine-rich peptides being of particular interest due to their unique properties [2]. Sea anemones possess cnidocytes with nematocysts specialized for producing and delivering toxins crucial for their survival in the marine ecosystem [3]. Activation of these nematocysts leads to the discharge of venom in response to mechanical or chemical stimuli, which is essential for both predation and defense [4], and which are regarded as a pharmacological tool or a reservoir for natural drugs [5]. Marine biotoxins exhibit enormous potential for the development of new drugs. So far, more than 20 drugs derived from the ocean have been approved for the market worldwide. For example, ω-Conotoxin MVIIA (Ziconotide, Prialt), which is a peptide derived from *Conus magus,* is used to relieve pain. The ShK peptide from the sea anemone *Stichodactyla helianthus* [6] is a potent blocker of voltage-gated potassium channels. Its derivative, ShK-186, is currently undergoing early clinical trials to treat T-cell autoimmune diseases such as multiple sclerosis by inhibiting the Kv1.3 channel [7]. Therefore, research on marine biotoxins is currently receiving great attention.

Sea anemone toxins produced by prickly cells are typically classified into multiple categories, including phospholipase A2; cytolysins; insulin-like peptides; protease inhibitors, such as serine protease inhibitors; and neurotoxic peptides affecting a range of ion channels, like voltage-gated sodium (Nav), calcium (Cav), and potassium (Kv) channels [8,9,10,11,12]. Sea anemone toxins notably engage in inflammatory and nociceptive processes [13,14], cellular membrane lysis [15], and the modulation of ionic channel dynamics, including the immunosuppressive effects of ShK domain peptides on the Kv1.3 channel [16].

The South China Sea boasts abundant sea anemone resources, including *Heteractis crispa* (*H. crispa*), *Exaiptasia diaphana* (*E. diaphana*), *Heteractis magnifica* (*H. magnifica*), *Macrodactyla doreensis* (*M. doreensis*), etc. At present, a large number of studies have reported the diversity of venom from *H. crispa*, *E. diaphana*, and *H. magnifica*, all of which have been systematically explored through transcriptomics owing to their rich peptides [17,18,19]. However, there are currently almost no reports on *M. doreensis*, and this study is the first to conduct a systematic transcriptomic analysis of this resource. *M. doreensis* is widespread in the Indo-Pacific region, particularly in the South China Sea, where it is very common. The preliminary research on the venom of *M. doreensis* proved its pharmacological anti-cancer, insecticidal, and analgesic activities. Therefore, it is particularly warranted to conduct in-depth research on the protein and peptide components in *M. doreensis*.

In the early study of sea anemone toxins, traditional methods such as the fractionation and purification of crude venom, as well as gene cloning through PCR primer design, were predominantly employed to discover novel sea anemone toxins. These separation and purification techniques were both laborious and time-consuming, with significant limitations. With the rapid development of high-throughput sequencing (HTS) technology, the limitations of traditional research techniques have been overcome. It has the advantages of high sequencing throughput, high sensitivity, low cost, and the ability to discover novel protein and peptide gene sequences. Therefore, this study used HTS to perform comprehensive transcriptome analysis on the tentacles and column of *M. doreensis* and constructed a putative protein and peptide database. Compared with emphasis of previous studies on the transcriptome of sea anemones, this study focuses on typical peptide families, predicts their 3D structures through AlphaFold2 modeling, and evaluates their potential activities through molecular docking, laying a solid foundation for the development of new marine drugs.

## 2. Results

### 2.1. Transcriptome Sequence Assembly

HTS has revealed that 49,077,330 and 45,623,592 paired-end short reads (150 bp in length) were obtained from the tentacles and column, respectively, with Q30 quality scores surpassing 91%. Subsequent to the removal of inferior reads and adapters, the refined dataset yielded a total of 93,660,778 clean reads, with clean data ratios averaging 98.90%. The de novo assembly process resulted in the generation of 287,880 unique sequences across both tissue samples, exhibiting a mean N50 length of 1499 bp (Table S1).

Analyses of the GC content in the assembled contigs and unigenes revealed percentages of 41.18% in tentacle samples and 38.90% in column samples. Functional annotation against multiple databases, including the Non-Redundant Protein Sequence Database (Nr), the Kyoto Encyclopedia of Genes and Genomes (KEGG), Clusters of Orthologous Groups for Eukaryotic Complete Genomes (KOG), and the Universal Protein Resource (UniProt), revealed a substantial portion of unigenes with functionalities yet to be ascribed. The most prominent categories within the KEGG pathways were metabolism and organismal systems, providing valuable insights into the metabolic capabilities of sea anemones and their interactions within the organismal systems.

GO categorization highlighted 85,897 unigenes across three domains: biological process, cellular component, and molecular function, with notable enrichment in cellular anatomical entities and cellular processes. KOG database annotations further distributed 19,922 and 17,159 unigenes across 25 functional classes, with general function prediction and signal transduction mechanisms being predominant. This comprehensive transcriptomic profiling dramatically enhances our understanding of *M. doreensis* at the molecular level, providing a broader biological and functional genomic context (Figure S1).

### 2.2. Family Classification of Putative Proteins and Peptides in the M. doreensis Transcriptome

**Figure 1:**
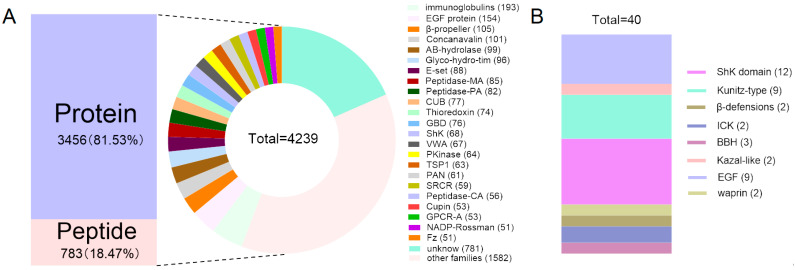
Putative protein and peptide families identified in the transcriptome of *M. doreensis*. (**A**) The proportion of putative proteins to peptides and classification diagram of families. (**B**) The number of putative peptides in representative families.

In this study, the extensive UniProt database has been utilized to identify these transcripts. The analysis revealed that putative proteins represent a predominant 81.63% (3456) of the transcripts, while putative peptides accounted for the remaining 18.37% (783), which were divided into 216 known families (Figure 1). The putative proteins were distributed across numerous families, the most prominent being the immunoglobulins and EGF proteins (Figure 1A). A total of 606 peptides were derived from the transcriptomic profiles of *M. doreensis*’ tentacles and its column, cataloged as MD-001 to MD-606 (Table S2). Based on the framework of cysteine, 40 peptides have been further screened and divided into eight families: ShK domain, Kunitz-type, β-defensins, ICK, BBH, Kazal-like, EGF, and waprin (Figure 1B).

### 2.3. Comparative Analysis of Putative Proteins and Peptides in Different Tissues of M. doreensis

**Figure 2:**
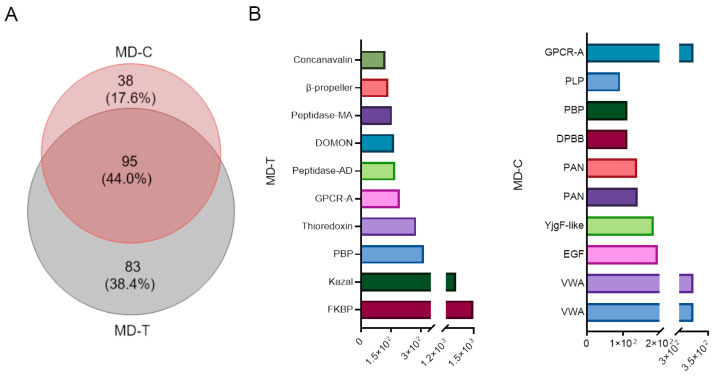
Comparison of transcripts from different parts of *M. doreensis*. (**A**) The Venn diagram of family classification relationship between MD-T and MD-C. (**B**) The ten most highly expressed transcripts from different tissues.

While exploring the transcriptomic landscapes of *M. doreensis*, distinct regional profiles reveal a diverse array of bioactive molecules. Cysteine-rich peptides are prevalent, suggesting a role in defensive or predatory behaviors. Comprehensive transcriptomic analysis of the tentacle and column regions of *M. doreensis* has revealed the relative classification of these putative proteins and peptides. The transcriptome in the tentacles of *M. doreensis* is divided into 133 families, while the transcriptome in the column is divided into 178 families, of which 95 families are shared (Figure 2A).

Data were converted to FPKM (fragments per kilobase of exon model per million mapped fragments) to quantify differentially expressed transcripts. The results revealed that FK506 binding protein (FKBP) and Kazal-like peptides exhibited the highest transcript levels in the tentacular region, whereas von Willebrand factor type A domain (VWA) was most prominent in the basal region (Figure 2B).

### 2.4. Sequence Analysis, Structure, and Target Prediction of Typical Putative Peptides

**Figure 3:**
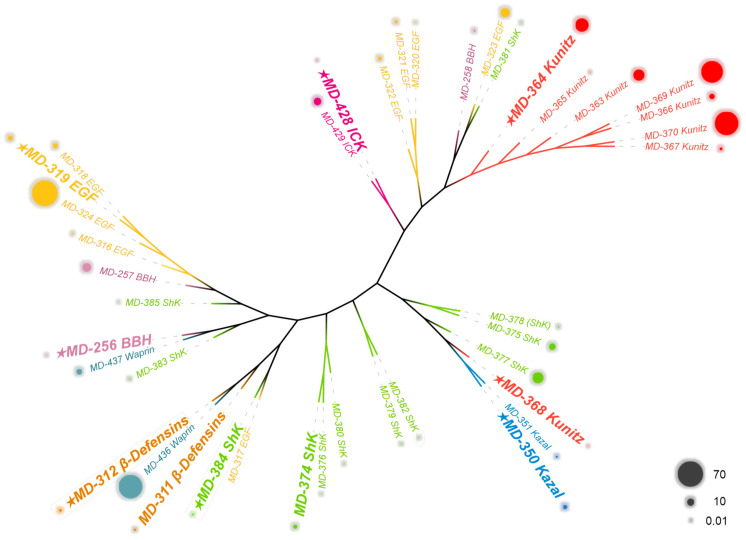
Phylogenetic and FPKM analysis of 40 putative peptides derived from *M. doreensis*. The tree was established using the ML approach. Sequences with the same color indicate peptides from the same family. The size of the outer circle of the sequence shows the FPKM.

The peptides derived from sea anemone venom serve as survival tools for sea anemones, exhibiting vast diversity and mostly classified based on cysteine patterns or structural features. They can act on a variety of ion channels or other receptors, such as Kv channels, Nav channels, and serine protease receptors [11,12,20]. This study conducted a comprehensive analysis of 40 putative peptides belonging to eight peptide families, including Kazal-like, EGF, waprin, and anemone types 1–5 potassium channel toxins, namely ShK domain, Kunitz-type, β-defensins, BBH, and ICK. Initially, a phylogenetic analysis was performed on 40 putative peptides, accompanied by a presentation of their corresponding FPKM (Figure 3). The results indicate that all putative peptides can be categorized into four major classes. While some of these classifications are consistent with cysteine pattern-based family classifications, there are significant deviations for the majority. Specifically, the β-defensins, ICK, Kazal-like, and Kunitz-type peptides (excluding MD-368), each form distinct branches, demonstrating strong evolutionary and affinity relationships consistent with classifications based on cysteine patterns. In contrast, ShK domain, EGF, waprin, and BBH peptides cluster into two or more branches. From the perspective of FPKM, Kunitz-type peptides exhibit the highest expression levels, followed by EGF peptides. Although a large number of putative peptides belong to the ShK domain, their expression levels are relatively low. The ShK domain peptides exhibit low amino acid sequence similarity and low FPKM values, indicating a high diversity of peptides in this family, but low individual expression levels. This suggests that the ShK domain peptides may possess a wide range of targets and activities.

#### 2.4.1. ShK Domain

ShK domain peptides also belong to the sea anemone type 1 potassium channel toxin family, and their representative peptide ShK consists of 35 amino acid residues intertwined with three disulfide bridges (Cys1-Cys6, Cys2-Cys4, Cys3-Cys5). These peptides are significant Kv channel inhibitors, predominantly targeting the type 1 channels [21,22]. Drugs used to treat autoimmune diseases typically produce systemic immune suppression, and ShK domain peptides can target the Kv1.3 channel to selectively inhibit CCR7-TEM lymphocytes and reduce this side effect [23].

In this study, eight representative peptides from known ShK structural domain peptides were selected for comparative analysis. ShK mainly inhibits Kv channels [24] and exerts some antibacterial effects [22]. BgK primarily targets the Kv channels (type 1), exhibiting potent inhibition of the Kv1.1 channel [25]. Its inhibitory effect on the remaining subtype channels is relatively weak; AsKs and HmK predominantly interact with the Kv1.2 channel [26,27], with the latter also influencing neurotransmitter release in animal models. AeK acts on the Kv channels (type 1) [28], while BCsTx1 and BCsTx2 focus their inhibitory actions on the Kv1.1 and Kv1.2 channels [29].

**Figure 4:**
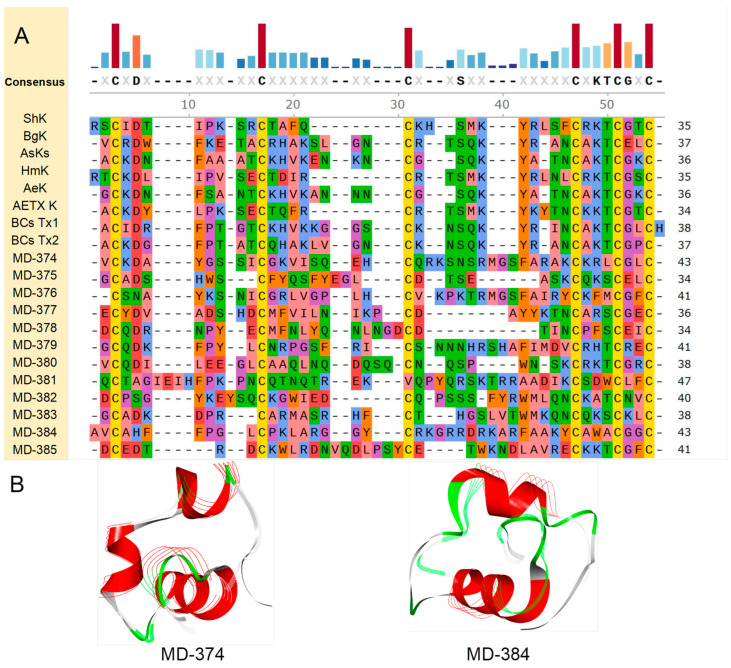
Deduced ShK domain transcripts from *M. doreensis*. (**A**) Multiple sequence alignment of known ShK domain peptides and corresponding putative mature peptides. Residues are shown by physicochemical properties, following their Zappo colors. The representative ShK domain peptides shown are ShK (Uniport: P29187; PDB: 1BEI), from the sea anemone *Stichodactyla helianthus*; BgK (Uniport: P29186; PDB: 1BGK), from the red warty sea anemone *Bunodosoma granuliferum*; AsKs (Uniport: Q9TWG1; AlphaFold: AF-Q9TWG1-F1), from the Mediterranean snakelocks sea anemone *Anemonia sulcata*; HmK (Uniport: O16846; AlphaFold: AF-O16846-F1) from the magnificent sea anemone *Heteractis magnifica*; AeK (Uniport: P81897; AlphaFold: AF-P81897-F1) from the beadlet anemone *Actinia equina*; BCsTx1/2 (Uniport: C0HJC2/C0HJC3; AlphaFold: AF-C0HJC2-F1/AF-C0HJC3-F1) from the sea anemone *Bunodosoma caissarum*. (**B**) The putative peptide models (flat representation) of the ShK domain predicted by AlphaFold2 (the line model that overlaps with them is the best peptide obtained in the model alignment).

Through amino acid sequence analysis of 12 homologous putative peptides (MD-374 to MD-385), it was determined that these peptides showed the highest sequence similarity with seven known peptides of the ShK domain: ShK corresponding to MD-380 (36.59%), BgK to MD-374 (44.19%), AsKs to MD-380 (47.37%), HmK to MD-382 (39.02%), AeK to MD-377 (44.74%), BCsTx1 to MD-384 (46.67%), and BCsTx2 to MD-377 (46.15%) (Figure 4A). Based on the amino acid sequence of each putative peptide, AlphaFold2 was used for modeling to predict their respective 3D structures. The predictive models uniformly adhere to the archetypal architecture of the ShK domain, characterized by intricate arrays of α-helix bundles and helix-turn (loop)-helix motifs. Notably, the α-helices, situated proximate to the N-terminus and C-terminus, display an almost perpendicular, parallel arrangement. Specifically, the model aligned based on eight databases, i.e., AlphaFold/Proteome, AlphaFold/Swiss-Prot, AlphaFold/UniProt50, BFMD, CATH50, GMGCL, MGnify-ESM30, and PDB100. The protein or peptide exhibiting the most structurally similar fragment to each of the putative peptides was identified. Notably, MD-374 structurally aligns most closely with U-actidoxin-Avd8d, derived from the snakelocks anemone *Anemonia viridis*, with a TM-score of 0.94897 and an RMSD of 0.51. Additional information pertaining to other putative peptides is appended in Table S4.

**Figure 5:**
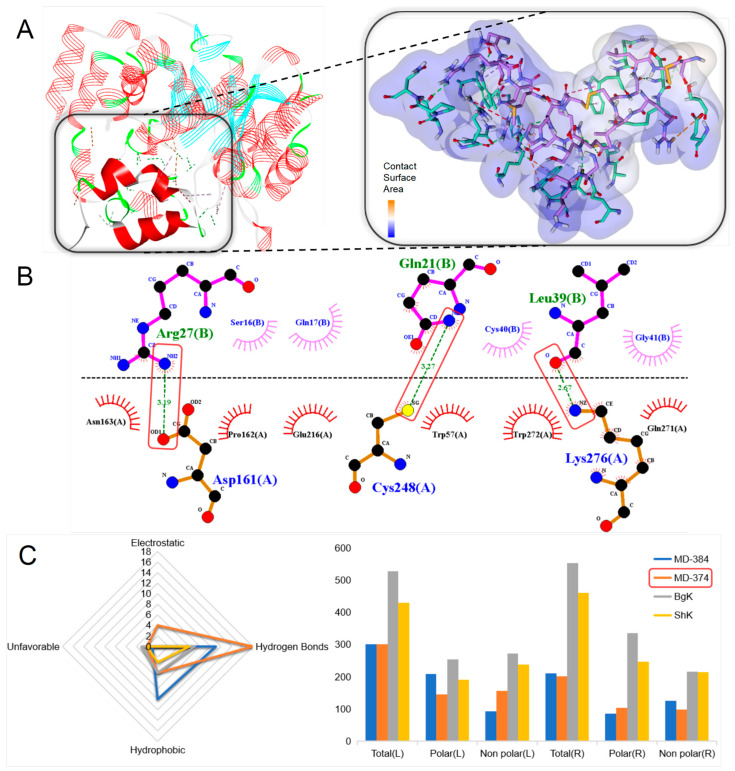
Molecular docking analyses revealed the binding interaction between the ShK domain peptides and the Kv1.3 channel. The green dotted lines indicate hydrogen bond interactions, the red dotted lines represent unfavorable interactions, the yellow dotted lines denote electrostatic interactions, and the pink dotted lines highlight hydrophobic interactions. (**A**) A docking pattern diagram illustrating MD-374 (flat representation) and the Kv1.3 channel (PDB: 7EJ1) (line representation), along with a more comprehensive 3D diagram displaying the docking locations and contact surface area of MD-374 (colored purple) and the Kv1.3 channel (colored green). (**B**) An eyelash (2D) diagram depicting the key docking sites of MD-374 (colored blue) and the Kv1.3 channel (colored green). (**C**) A radar chart illustrating the number of non-bond interactions between ShK domain peptides and the Kv1.3 channel, along with a 3D surface plot depicting the contact surface area. Ligand (L); receptor (R).

The Kv1.3 channel plays an important role in the pathogenesis of autoimmune diseases and is also associated with analgesia and anti-cancer effects [23,24]. A large-scale molecular docking of 20 ShK domain peptides with the Kv1.3 channel was conducted to demonstrate their potential interactions and biological effects on the potassium channels [30]. MD-374 was found to form the most stable peptide–protein complex, exhibiting the potential to form extensive non-covalent bonds with the target protein (Figure 5A). According to the prediction result, the complex has a total of 27 favorable non-covalent bonds, of which 18 are hydrogen bonds, such as Asp161-Arg27, Cys248-Gln21, and Lys276-Leu39 (Figure 5B). The contact surface area between the ligand and the receptor is 526.64 and 552.76, respectively. Compared with the molecular docking results of other known ShK domain peptides, MD-374 may exhibit more effective inhibitory activity on the Kv1.3 channel.

#### 2.4.2. Kunitz-Type

Kunitz-type peptides derived from sea anemones belong to the sea anemone type 2 potassium channel toxin family and possess dual functional capacities. They not only inhibit the Kv1.2 channel but also act as serine protease inhibitors. The configuration of the three disulfide bonds within the sea anemone Kunitz-type peptides follows a specific arrangement, i.e., Cys1-Cys6/Cys2-Cys4 and Cys3-Cys5, as indicate by the cysteine pattern C-C-C-C-CX3C. The first two disulfide bridges play a crucial role in maintaining the conserved nature of the tertiary structure of the domain, while the third bond is critical for stabilizing the active binding site [31].

This study selected 10 representative peptides from known Kunitz-type peptides for comparative analysis. The peptides designated as AsKC1/2/3 are documented to exhibit inhibitory actions against serine proteases, as well as suppression of the Kv channels, primarily the Kv1.2 channel [26]. AXPI-I is characterized by its potent inhibition of trypsin, with a moderate level of inhibition exhibited towards plasmin and only a weak effect on chymotrypsin, elastase, and the metalloprotease thermolysin. Notably, AXPI-I does not display inhibitory activity against the Kv channels [32]. The peptide DTX-α, although homologous to serine proteases, does not confer inhibitory effects upon serine proteases; instead, it is capable of obstructing the Kv channels, specifically the Kv1.1, Kv1.2, and Kv1.6 channels [33,34]. SHTXIII, another peptide in this class, can inhibit both serine proteases and the Kv channels [35].

**Figure 6:**
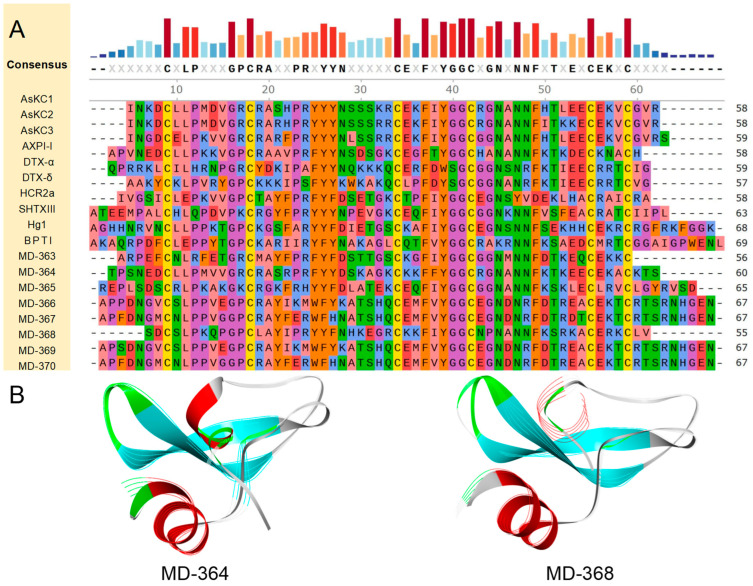
Deduced Kunitz-type transcripts from *M. doreensis*. (**A**) Multiple sequence alignment of known Kunitz-type peptides and corresponding putative mature peptides. The representative Kunitz-type peptides shown are BPT1 (Uniport: P00974), from the Bovine *Bos taurus*; AsKC1/2/3 (Uniport: Q9TWG0/Q9TWF9/Q9TWF8; AlphaFold: AF-Q9TWG0-F1/AF-Q9TWF9-F1/AF-Q9TWF8- F1), from the Mediterranean snakelocks sea anemone *Anemonia sulcata*; AXPI-I (Uniport: P81547; AlphaFold: AF-P81547-F1), from the sea anemone *Anthopleura aff. Xanthogrammica*; DTX-α/δ (Uniport: P00980/P00982; PDB: 1DTX/AlphaFold: AF-P00982-F1), from the Eastern green mamba or Naja angusticeps *Dendroaspis angusticeps*; SHTXIII (Uniport: B1B5I8; AlphaFold: AF-B1B5I8-F1), from the saddle carpet anemone, or Haddon’s sea anemone *Stichodactyla haddoni*. (**B**) The Kunitz-type putative peptide models (flat representation) predicted by AlphaFold2 (the line model that overlaps with them is the best peptide obtained in the model alignment).

Through amino acid sequence analysis, a suite of nine putative peptides, MD-363 to MD-371, was identified as homologous to AsKC-1, DTX1, and SHTXIII, with a conserved cysteine pattern of CX8CX15CX7CX12CX3C. The results of the homology comparison indicated that MD-364 exhibited the highest similarity and identity among the aforementioned peptides, with an overall similarity and identity of approximately 56.26% and 49.20%, respectively. Notably, its similarity to AsKC1/2 and AXPI-I exceeded 70.00%, with the identity surpassing 65.00% (Figure 6A).

Based on the amino acid sequence of each putative peptide, AlphaFold2 was used for modeling to predict their respective 3D structures (Figure 6B). All predictive models possess the typical structure of Kunitz-type peptides, which is composed of a helix-loop-sheet-turn-sheet-loop-helix configuration extending from the N-terminus to the C-terminus. This architecture includes a β-hairpin and features a preserved, compact hydrophobic core inherent to the α-β-β motif. Specifically, the protein or peptide with the most similar structural fragment to each putative peptide was discovered. MD-364 is structurally most similar to U-actitoxin-Avd3j from the Mediterranean snakelocks sea anemone *Anemonia sulcata*, with a TM-score of 0.95746, and an RMSD of 0.74. Other putative peptide-related information is attached in Table S4.

**Figure 7:**
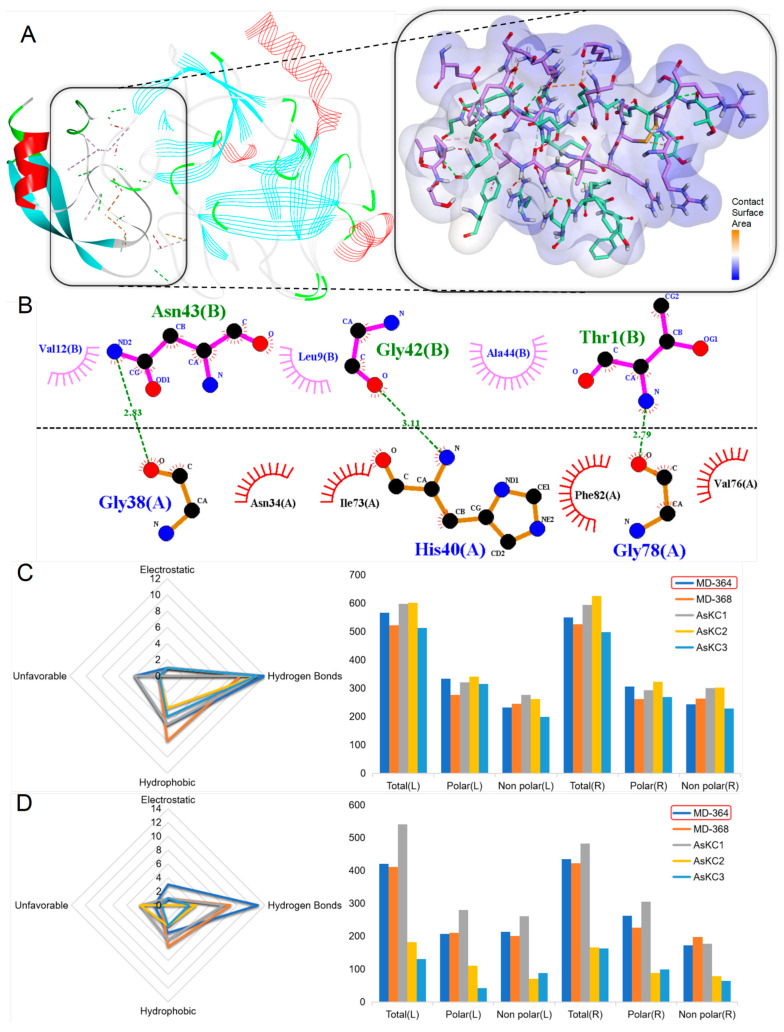
Molecular docking analyses revealed the binding interaction between Kunitz-type peptides and the serine proteinase receptor. (**A**) A docking pattern diagram illustrating MD-364 (flat representation) and the serine proteinase receptor (PDB: 5PTP) (line representation), along with a more comprehensive 3D diagram displaying the docking locations and contact surface area of MD-364 (colored purple) and the serine proteinase receptor (colored green). (**B**) An eyelash diagram depicting the key docking sites of MD-364 (colored blue) and the serine proteinase receptor (colored green). (**C**,**D**) A radar chart illustrating the number of non-bond interactions between Kunitz-type peptides and the serine proteinase receptor/Kv1.2 channel, along with a 3D surface plot depicting the contact surface area.

Due to the dual inhibitory properties of Kunitz-type peptides on the Kv channels and serine proteases, a large-scale molecular docking study was conducted to elucidate the potential interactions and biological efficacy of 16 Kunitz-type peptides with the Kv1.2 channel and serine proteases. The Kv1.2 channel is primarily implicated in neurological disorders such as epilepsy, intellectual disabilities (ID), attention deficit/hyperactivity disorder (ADHD), autism spectrum disorder (ASD), pain, and autoimmune conditions [36]. Serine proteases are associated with inflammation and cancer. In the screenings of putative peptides in this family, MD-364 emerged as the peptide that forms the most robust peptide–protein complex with serine proteases, showing a potential capacity for extensive non-covalent bonds with target proteins (Figure 7A). Based on predictive analyses, this complex boasts a total of 19 favorable non-covalent bonds, 12 of which are hydrogen bonds, including those between Gly78 and Thr1, His40 and Gly42, as well as Gly38 and Asn43 (Figure 7B). The contact surface area between the ligand and the receptor is 549.64 and 549.64, respectively. MD-364 outperformed all other putative peptides within the same family and even surpassed known peptides, hinting at its superiority. Consequently, MD-364 may demonstrate a stronger inhibitory influence against serine proteases compared to that of other Kunitz-type peptides. With respect to the docking outcomes involving the Kv1.2 channel, within its family, MD-364 remains the peptide predicted as the most likely to form a stable complex with the receptor, thereby implying a possible inhibitory influence on the Kv1.2 channel (Figure 7C).

#### 2.4.3. β-Defensins

The β-defensins discovered in sea anemones are also classified as comprising the sea anemone type 3 potassium channel toxin family. They typically consist of approximately 40 amino acids and display an essential pattern of disulfide linkage (Cys1-Cys5, Cys2-Cys4, and Cys3-Cys6) crucial for structural stability [37]. β-defensins display selective activities against Nav channels (types 1, 2, and 4), the Kv1.3 channel, ASICs, and TRPV1, modulating physiological processes pivotal to host defense [38,39].

This study selected five representative peptides from known β-defensins peptides for comparative analysis. Am II is a low molecular weight neurotoxin that causes paralysis when injected into crabs, suggesting a potential inhibitory or blocking effect on the Nav channels [40]. BDSI and BDSII are inhibitors of the Kv channels, predominantly affecting type 3 [41,42]. BDSI prolongs the opening duration of the Nav channels, explicitly targeting the Nav1.7 channel, resulting in a hypotensive effect [40]. BCIV is another peptide neurotoxin that affects the activity of the Nav channels without impacting the Kv channels [39]. APETx1 primarily affects the ERG potassium ion channels and the sodium ion channels. Its mechanism of action involves upregulating the voltage required to activate the channels and limiting the amplitude of their action potentials [43,44].

**Figure 8:**
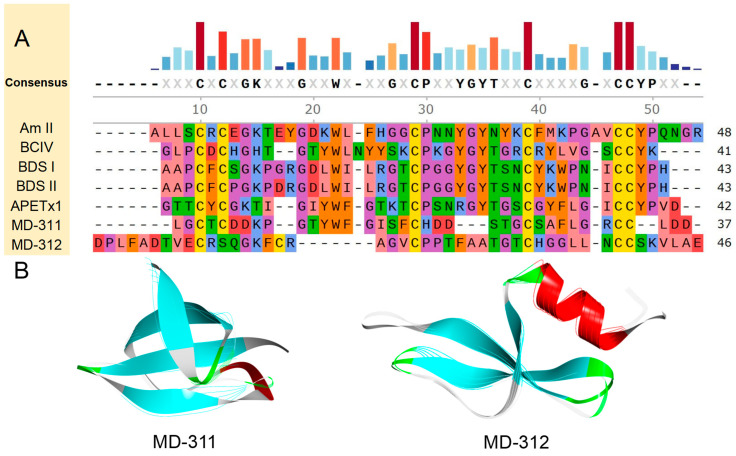
Deduced β-defensins transcripts from *M. doreensis*. (**A**) Multiple sequence alignment of known β-defensins peptides and corresponding putative mature peptides. The representative β-defensins peptides shown are Am II (Uniport: P69930; AlphaFold: AF-P69930-F1), from the sea anemone *Antheopsis maculata*; BCIV (Uniport: P84919; AlphaFold: AF-P84919-F1), from the sea anemone *Bunodosoma caissarum*; BDSI/II (Uniport: P11494/P59084; PDB:1BDS/AlphaFold: AF-P59084-F1), from the Mediterranean snakelocks sea anemone *Anemonia sulcata*; APETx1 (Uniport: P61541; PDB: 1WQK), from the green aggregating anemone, or Actinia elegantissima *Anthopleura elegantissima*. (**B**) The putative peptide models (flat representation) of β-defensins predicted by AlphaFold2 (the line model that overlaps with them is the best peptide obtained in the model alignment).

Through amino acid sequence analysis, two putative peptides affiliated with the β-defensins family were identified, namely MD-311 and MD-312, exhibiting a cysteine pattern characteristic of CXC-C-C-CC. A subsequent homology comparison revealed that MD-311 showed the highest similarity with BCIV, at 38.10%, and the highest sequence identity with APETx1, at 33.33%. Additionally, MD-312 displayed the highest similarity with BCIV, at 35.29%, and the highest sequence identity with BDSI, at 26.42% (Figure 8A).

Based on the amino acid sequence of each putative peptide, AlphaFold2 was used for modeling to predict their respective 3D structures. The prediction results show that all models exhibit typical structural features, predominantly displaying multiple β-fold composition. All predictive models prominently featured a β-barrel, emphasizing the presence of a highly conserved structural core β-fold, which is characteristic of the β-defensins family. Specifically, the protein or peptide with the most similar structural fragment to each putative peptide was discovered. MD-312 is structurally most similar to gallinacin-10, from chicken *Gallus gallus*, with a TM-score of 0.90943, and an RMSD of 0.97. Other putative peptides-related information is attached in Table S4.

**Figure 9:**
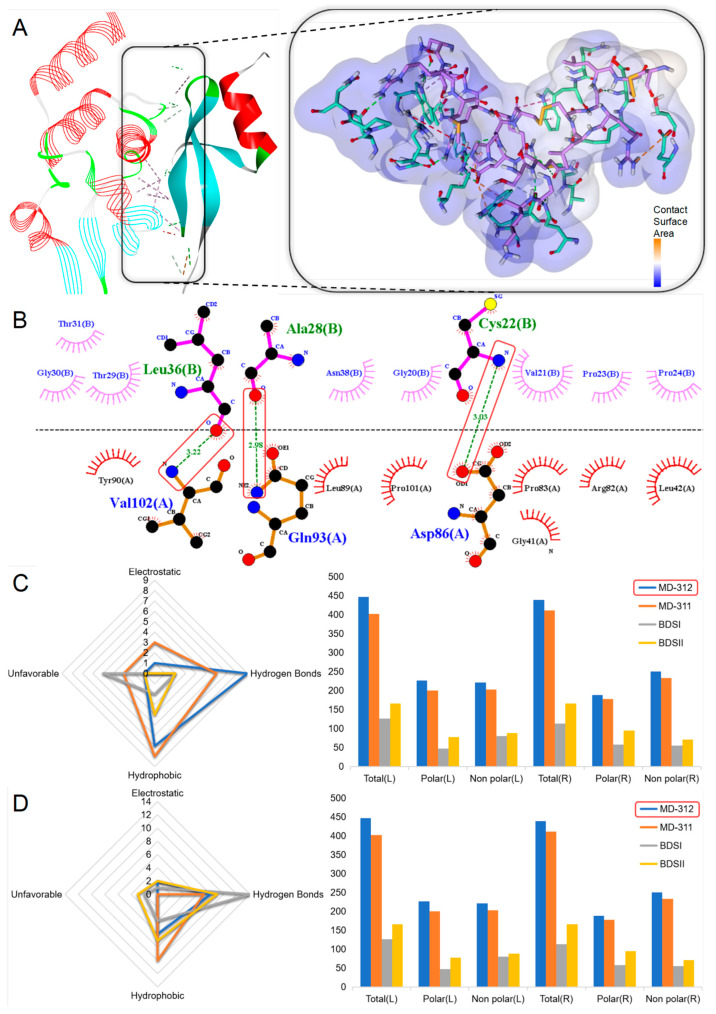
Molecular docking analyses revealed the binding interaction between β-defensins peptides and the Kv1.2 channel. (**A**) A docking pattern diagram illustrating MD-312 (flat representation) and the Kv1.2 channel (PDB: 1QDV; A chain) (line representation), along with a more comprehensive 3D diagram displaying the docking locations and contact surface area of MD-312 (colored purple) and the Kv1.2 channel (colored green). (**B**) An eyelash diagram depicting the key docking sites of MD-312 (colored blue) and the Kv1.2 channel (colored green). (**C**,**D**) A radar chart illustrating the number of non-bond interactions between the β-defensins peptides and the Kv1.2/Nav1.7 channels, along with a 3D surface plot depicting the contact surface area.

Due to the role of β-defensins peptides as Kv channels blockers, along with their effects on inhibiting or prolonging the opening times of the Nav channels, a comprehensive molecular docking study was conducted involving seven β-defensins peptides with Kv1.2 and Nav1.7 channels to explore their potential interactions and biological efficacy. Both the Kv1.2 and Nav1.7 channels are involved in the mechanisms underlying pain responses, with the Nav1.7 channel encoded by the SCN9A gene and primarily located in the dorsal root ganglion neurons, making it a crucial target for pain management. Loss of function in the Nav1.7 channel results in insensitivity to pain [45]. Within the β-defensins peptides identified from our transcriptomic screening, MD-312 was found to form the most stable peptide–protein complex with both the Kv1.2 and Nav1.7 channels, exhibiting substantial potential for extensive non-covalent bonds with these target proteins (Figure 9A). Specifically, MD-312 was predicted to form nine hydrogen bonds with the Kv1.2 channel, e.g., between Asp86 and Cys22, Gln93 and Ala28, and Val102 and Leu36 (Figure 9B). In total, there were 17 favorable non-covalent bonds, which surpassed the number for the comparative peptides in this aspect. In terms of interactions with the Nav1.7 channel, MD-312 also demonstrated optimal performance. It is expected to form 16 favorable non-covalent bonds, including 8 hydrogen bonds, surpassing other peptides in terms of ligand–receptor contact surface area, without any unfavorable interactions (Figure 9C). These results suggest that MD-312 has exceptionally high biological potential for targeting both the Kv1.2 and Nav1.7 channels.

#### 2.4.4. BBH

The BBH peptides discovered in sea anemones are also classified as comprising the sea anemone type 3 potassium channel toxin family. These peptides, commonly found in sea anemones, are composed of cysteine-rich chains, typically ranging from 25 to 40 amino acid residues in length. They are distinguished by the formation of two discrete disulfide bonds between four cysteine residues, manifesting an intramolecular bonding configuration typified as Cys1-Cys3 and Cys2-Cys4. Such a disulfide bond framework is identified by the cysteine pattern CX2C-C [46].

This study selected six representative peptides from the known BBH peptides for comparative analysis. The peptide SHTX-I/2 has been identified as an inhibitor of the Kv channels [45]. In contrast, Ugr 9a-1 is known to exert an influence on the ASIC3 channel, imbuing it with anti-inflammatory and analgesic characteristics that are attributed to its distinctive spatial structure and mechanism of action [47]. The peptide BCg III 23.41 is speculated to inhibit the function of the Kv channels [48]. Furthermore, Tau-AnmTX Ms 9a-1 has been shown to act as an agonist to mammalian TRPA1 and to demonstrate anti-inflammatory and analgesic activities in vitro [49].

Through amino acid sequence analysis, three putative peptides affiliated with the BBH, denoted as MD-256, MD-257, and MD-258, were identified. It was determined that MD-256 shows the highest sequence similarity and identity with SHTX-I/2, at 35.00% and 22.50%, respectively. The similarity levels with the other four peptides are approximately 30%, while the similarity between MD-257 and MD-258 and these peptides is relatively low (Figure S2A).

Based on the amino acid sequence of each putative peptide, AlphaFold2 was used for modeling to predict their respective 3D structures. All predictive models inherently possess the β-hairpin motif, emphasizing the consistent structural essence that characterizes the BBH family.

Since BBH peptides are Kv channel blockers, a large-scale molecular docking of a total of nine BBH peptides with the Kv1.1 channel was conducted to demonstrate the potential interactions and biological potential of these peptides with the Kv1.1 channel. The Kv1.1 channel has been associated with epilepsy, multiple sclerosis, episodic ataxia, and cardiorespiratory dysregulation [50]. Among the BBH peptides identified in our transcriptome screening, MD-256 was found to form the most stable peptide–protein complex, exhibiting the potential to form numerous hydrogen bonds (Thr83-Cys10, etc.) and non-covalent (Arg67-Asp9 etc.) interactions with the target protein (Figure S3A,B). Specifically, it showed similar counts of anticipated favorable non-covalent bonds when compared with SHTX-I/2 (14; 16) but presented fewer unfavorable interactions (1 vs. 2). Additionally, the ligand-receptor interface areas were found to be comparable between the two peptides (Figure S3C). These results suggest that MD-258 may have an inhibitory impact on the Kv1.1 channel similar to that of SHTX-I/2.

Among the BBH peptides identified in our transcriptome screening, MD-256 has been identified as forming the most stable peptide–protein complex, exhibiting the potential to form extensive non-covalent bonds with the target protein (Figure S3A,B). Specifically, it showed a slightly lower number of hydrogen bonds than did SHTX-I/2 (10 vs. 12), while displaying a reduced number of unfavorable interactions (0 vs. 2). Furthermore, an analysis revealed a similarity in the ligand and receptor contact surface area between the two peptides (Figure S3C). These findings imply that MD-256 may possess an inhibitory effect on the Kv1.1 channel, akin to that exhibited by SHTX-I/2.

#### 2.4.5. ICK

The ICK peptides discovered in sea anemones are also classified as comprising the sea anemone type 5 potassium channel toxin family. They are predominantly characterized by their approximately 50 amino acids. These sequences are stabilized through a network of internal pairings among eight cysteine residues, resulting in four discrete intramolecular disulfide bridges. It is posited that a distinct disulfide bonding arrangement prevails within these peptides, involving the linkages Cys1-Cys4, Cys2-Cys5, Cys3-Cys8, and Cys6-Cys7, which establish a framework as C-C-CC-CX3C-C-C [51,52].

This study selected four representative peptides from known ICK peptides for comparative analysis. PP III is recognized as an inhibitor of the Nav and Cav channels. PP III is characterized by a voltage-independent mechanism that obstructs the conductivity of ion channels by blocking the extracellular vestibular pores, which can lead to fatal paralysis of the target insect. The efficacy of PP III in binding to and inhibiting sodium and calcium channels is likely integral to its formidable insecticidal properties [53]. BcsTx3 is known to target the Kv channels, predominantly interacting with the Kv1 subtypes, an intervention that provokes paralytic effects in crabs [51]. Similarly, NvePTx1 and MsePTx1, peptides that closely mirror the primary amino acid composition of BcsTx3 and PP III, are identified as neurotoxins. They are speculated to potentially inhibit the Kv channels [54,55].

**Figure 10:**
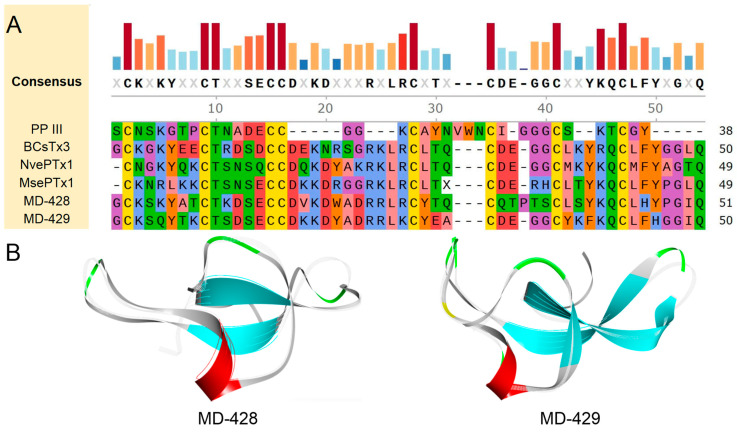
Deduced ICK family transcripts from *M. doreensis*. (**A**) Multiple sequence alignment of known ICK peptides and corresponding putative mature peptides. The representative ICK peptides shown are PP III (Uniport: P49268; PDB:2M36), from the trap-door spider, or Aptostichus schlingeri *Apomastus schlingeri*; BcsTx3 (Uniport: C0HJC4; AlphaFold: AF-C0HJC4-F1), from the saddle carpet anemone, or Haddon’s sea anemone *Bunodosoma caissarum*; NvePTx1 (Uniport: A7RMN1; AlphaFold: AF-A7RMN1-F1), from the starlet sea anemone *Nematostella vectensis*; MsePTx1 (Uniport: P0DMD7), from the brown or frilled sea anemone *Metridium senile*. (**B**) The putative peptide models (flat representation) of ICK predicted by AlphaFold2 (the line model that overlaps with them is the best peptide obtained in the model alignment).

Through amino acid sequence analysis, two putative peptides, designated MD-428 and MD-429, were identified. They exhibit a cysteine pattern characteristic of C-C-CC-CX3C-C-C. It has been determined that MD-428 shares notable similarity and identity with Bcs4a, with values of 70.59% and 56.86%, respectively. Furthermore, MD-429 showed the highest sequence similarity and identity with NvePTx1, with values of 72.00% and 60.00%, respectively (Figure 10A).

Based on the amino acid sequence of each putative peptide, AlphaFold2 was used for modeling to predict their respective 3D structures (Figure 10B). All prediction models include the typical structure of ICK peptides, which is composed of a helix-loop-sheet-loop-sheet configuration extending from the N-terminus to the C-terminus. Central to this conformation is a conserved hydrophobic core, characterized by an α-β-β motif. This conservation in structure suggests the potential for similar functional properties among these peptides. Specifically, the protein or peptide with the most similar structural fragment to each putative peptide was discovered. MD-429 is structurally the most similar to NvePTx1, from the starlet sea anemone *Nematostella vectensis*, with a TM-score of 0.84807 and an RMSD of 2.30. Other putative peptides-related information is attached in Table S4.

**Figure 11:**
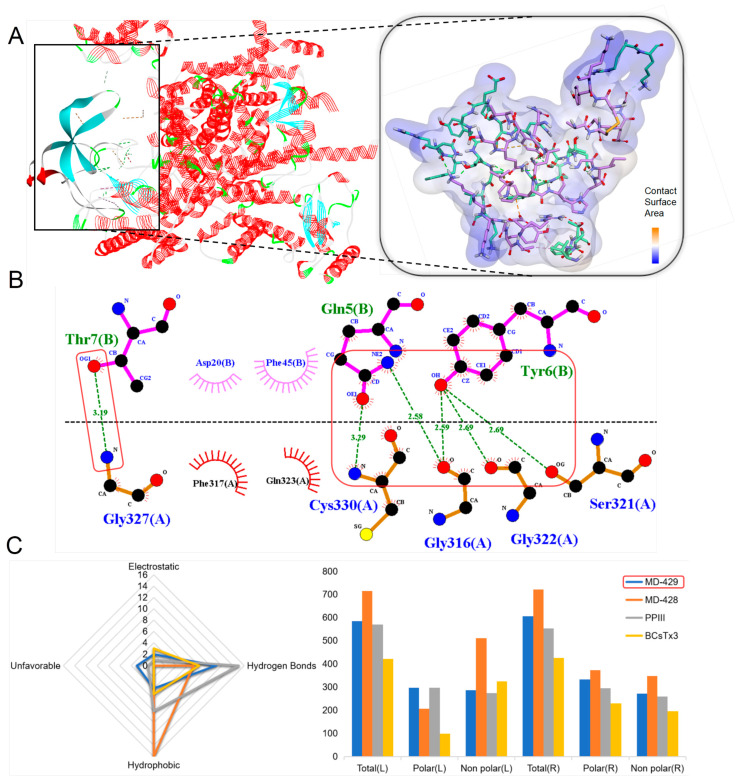
Molecular docking analyses revealed the binding interaction between ICK peptides and the Nav1.7 channel. (**A**) A docking pattern diagram illustrating MD-429 (flat representation) and the Nav1.7 channel (PDB: 7W9L; A chain) (line representation), along with a more comprehensive 3D diagram displaying the docking locations and contact surface area of MD-429 (colored purple) and the Nav1.7 channel (colored green). (**B**) An eyelash diagram depicting the key docking sites of MD-429 (colored blue) and the Nav1.7 channel (colored green). (**C**) A radar chart illustrating the number of non-bond interactions between ICK peptides and the Nav1.7 channel, along with a 3D surface plot depicting the contact surface area.

Since ICK peptides are Nav1.7 channel inhibitors, a molecular docking of a total of six ICK peptides with the Nav1.7 channel was conducted to demonstrate the potential interactions and biological potential of these peptides with the Nav1.7 channel. Among the ICK peptides identified in our transcriptome screening, MD-429 was found to form the most stable peptide–protein complex, exhibiting the potential to form extensive non-covalent bonds with the target protein (Figure 11A). Specifically, MD-429 is predicted to form 17 favorable non-covalent bonds with the receptor, including 11 hydrogen bonds, e.g., Gly327-Thr7; Cys330-Gln5; Ser321-Try6 (Figure 11B). The contact surface area between the ligand and the receptor was measured to be 583.35 and 605.31, respectively (Figure 11C). Compared with the known peptides PPIII and BCsTx3 within the same family, MD-429 exhibits a higher number of hydrogen bonds than BCsTx3, yet a lower number compared to PPIII. Furthermore, the contact surface area of the ligand and the receptor for MD-429 is greater than for either PPIII and BCsTx3. Consequently, it is speculated that MD-429 may possess a certain inhibitory effect on the Nav1.7 channel.

#### 2.4.6. Kazal-like

In the peptides of sea anemones, serine protease inhibitors predominantly fall under Kazal-like and Kunitz-type categories. We mainly introduce Kazal-like peptides here. This type of peptide is a single-chain peptide rich in cysteine residues in sea anemones, generally composed of about 50 amino acid residues. It has three pairs of disulfide bonds formed by six cysteine residues that are cross-linked with each other. The connection positions of the disulfide bonds within the molecule are Cys1-Cys5, Cys2-Cys4, and Cys3-Cys6, forming the cysteine pattern CX3C-C-C-C-C-C [56,57,58].

This study selected three representative peptides from known Kazal-like peptides for comparative analysis (AEI, TATI, and H3G689). Amino acid sequence analysis has revealed two putative peptides, MD-350 and MD-351, which exhibit close alignment with AEI, TATI, and H3G689, thereby highlighting the conserved structural motifs characteristic of this family of serine protease inhibitors (Figure S4A). Based on the amino acid sequence of each putative peptide, AlphaFold2 was used for modeling to predict their respective 3D structures. All prediction models possess the basic structural framework characteristics of Kazal-like peptides, as well as a conservative β-hairpin motif and a hydrophobic core α-β-β motif (Figure S4B). Since Kazal-like peptides are serine protease inhibitors, a molecular docking of a total of four Kazal-like peptides with the serine proteinase receptor was conducted to demonstrate the potential interactions and biological potential of these peptides with the serine proteinase receptor. Among the Kazal-like peptides identified in our transcriptome screening, MD-350 was found to form the most stable peptide–protein complex. Predictions indicate the formation of 13 favorable non-bonding interactions, comprising 10 hydrogen bonds, akin to the outcomes observed for AEI and TATI. Consequently, MD-350 potentially possesses a certain inhibitory effect on serine protease (Figure S5A).

#### 2.4.7. EGF

Most EGF peptides are composed of around 50 amino acids, predominantly found within animal proteins. They contain three pairs of conserved disulfide bonds, typically formed between cysteine residues at the Cys1-Cys3, Cys2-Cys4, and Cys5-Cys6 positions. The defining architectural element of this family is a central double-stranded β-sheet, bordered by the Cys1-Cys3 and Cys2-Cys4 regions, which gives rise to a β-hairpin structure [59,60].

This study selected seven representative peptides from known EGF peptides for comparative analysis (Mg1a, Gigt I, Gigt 4, BCs III 15.09, SHTX-5, human EGF, and mouse EGF). Through amino acid sequence analysis, nine putative peptides, designated MD-316 through MD-324, were identified. The analysis results indicate that Gigt I, Gigt 4, BCs III 15.09, and SHTX-5 exhibit the highest sequence similarity and identity (Figure S6A). Based on the amino acid sequence of each putative peptide, AlphaFold2 was used for modeling to predict their respective 3D structures. All prediction models encompassed β-sheets and an α-helix, emphasizing a consistently preserved structural core featuring α-β-β motifs, characteristic of EGF peptides. Since EGF peptides are epidermal growth factor receptor (EGFR) inhibitors, a molecular docking of all the above EGF peptides with EGFR was conducted to demonstrate the potential interactions and biological potential of these peptides with EGFR. Among the EGF peptides identified in our transcriptome screen, MD-323 was found to form the most stable peptide–protein complex, and MD-323 was discovered to constitute the most robust peptide–protein complex, with the prediction of the formation of 11 favorable non-covalent bonds, among which 10 are hydrogen bonds, resembling the outcomes observed for human EGF. Consequently, MD-323 potentially exhibits a certain inhibitory effect on EGFR (Figure S7A).

#### 2.4.8. Waprin

The waprin peptides represent a class of cationic peptides that are rich in disulfide bonds. They are distinguished by eight cysteine residues forming four intramolecular disulfide bridges, which give rise to the so-called four-disulfide core. These conserved cysteine residues are crucial for the formation of the disulfide bonds, and they typically follow the arrangement Cys1-Cys6, Cys2-Cys7, Cys3-Cys5, and Cys4-Cys8, conforming to the cysteine pattern C-C-C-C-CCX3CX3C [61,62].

This study selected four representative peptides from known waprin peptides for comparative analysis (omwaprin-a, supwaprin-a-like, supwaprin-a, and nawaprin). The homology analysis of the primary structure confirmed that the MD-436 and MD-437 putative peptides belong to the waprin family. MD-437 exhibits the highest sequence similarity and identity to the supwaprin-a-like peptide (Figure S8A). Based on the amino acid sequence of each putative peptide, AlphaFold2 was used for modeling to predict their respective 3D structures. All predictive models exhibited a fundamental structural framework characteristic of waprin peptides, which includes a sequence of loop, α-helix, β-turn, β-sheet, and β-sheet from the N-terminus to the C-terminus (Figure S8B). Furthermore, all of these models consistently featured a conserved β-hairpin motif, as well as a hydrophobic core α-β-β motif.

## 3. Discussion

In this study, HTS was used to conduct a comprehensive transcriptomic analysis of the tentacles and column of *M. doreensis* to explore the distribution characteristics and diversity of proteins and peptides, thereby establishing a functional database of putative proteins and peptides. Transcriptomics and bioinformatics were deeply combined to predict the family, 3D structure, and potential activity of putative peptides based on the primary (amino acid sequence analysis), secondary and tertiary (AlphaFold2 modeling), and quaternary structures (molecular docking) of the protein in order to screen putative peptides with potentially high activity.

With the aid of the UniProt database and a focus on primary structure homology, we achieved a comprehensive alignment of amino acid sequences. This database contains a total of 4239 sequences of putative proteins and peptides, which were divided into 216 known families. Most putative protein components matched those of immunoglobulins and a spectrum of protein kinases. The putative peptide components were related to the ShK domain, EGF, Kazal-like, β-defensins, Kunitz-type, and insulin-like peptides, echoing the characteristic venom components commonly identified in a multitude of known sea anemones [63]. Our data shows that within the tentacles, the prevalence of the primary constituents of sea anemone venom, such as the ShK domain, EGF, Kazal-like, and Kunitz-type peptides, is markedly higher than that noted in the column. Conversely, the immunoglobulin content in this column is more than twice that of the tentacles. This distribution pattern suggests a functional difference, in which the tentacles are usually used as hunting tools for sea anemones, injecting peptides with various biological activities such as paralysis and lethality into the prey through stinging cells, mainly related to predation behaviors [21,22,23,24,25,26,27,28,29]. The column is more focused on protective mechanisms, where the high level distribution of immunoglobulin plays a crucial role in resisting invading pathogens or foreign antigens [64].

The four common sea anemones in the South China Sea are *M. doreensis*, *H. crispa*, *E. diaphana*, and *H. magnifica.* Transcriptomic analysis shows that their peptide proportions are 18.7%, 9%, 27.02%, and 13.46%, respectively. They contain 12, 11, 11, and 13 ShK domain peptides; 7, 7, 10, and 12 Kunitz-type peptides; and 2, 11, 5, and 23 β-defensins peptides in the typical peptide families [17,18,19]. Through the above comparative analysis, it was discovered that teh ShK domain and Kunitz-type peptides are highly distributed in the sea anemones mentioned above, indicating that these two may be important peptides for the survival of sea anemone species. The high distribution of β-defensins peptides only noted in *H*. *magnifica* suggests that different sea anemones also exhibit diversity in their selection of peptides during their own evolution. The ShK domain peptides exhibit various biological activities, such as analgesia and lethality, and are more focused on competition and hunting [21,22,23,24], while the β-defensins peptides play a crucial role in immune responses and are more focused on defense and self-protection [38,39,40,41], and the Kunitz-type peptides have dual functions [32,33,34,35]. Furthermore, a phylogenetic analysis was conducted by integrating the ShK domain peptides, Kunitz-type peptides, and β-defensins peptides of these four sea anemones (Figure S9). In regards to the ShK domain peptides, *M. doreensis* is more closely related to *E. diaphana*.

Extensive identification of putative peptides across different regions revealed highly diverse peptides, particularly those forming multiple disulfide bonds. Based on amino acid sequence homology, this study identified 40 putative peptides across eight families, including the ShK domain, Kunitz-type, and Kazal-like peptides, among others. Our initial screening for these putative peptides was based on the homology to known peptide sequences within the same family. The 3D structure characterized intrinsic structural motifs, providing a clearer understanding of the molecular frameworks of these putative peptides. Subsequently, the binding strength of the quaternary structure was predicted using molecular docking technology and was compared with that of known analogues to evaluate the activity of the putative peptide. This visualization approach demonstrates the application of computational biology and structural bioinformatics in investigating molecular interactions and predicting conformations within biological functions.

The molecular underpinnings unveil an intricate map of peptide sequences spanning multiple families, each with unique biological characteristics and potential pharmacological applications. We elaborate on the relevant findings within eight major peptide families, including the ShK domain, Kunitz-type, β-defensins, and ICK. First, cysteine residues within the amino acid sequence were utilized as the primary criterion for categorizing peptides due to the fundamental role that the primary structure plays in predicting putative peptide structure. Highly conserved among various peptide families, cysteine residues are crucial for the formation of disulfide bonds that confer stability to the peptide’s tertiary structure, maintaining specific conformations and spatial arrangements which are critical for determining peptide stability and functional specificity. The distribution characteristics and resultant effects of other amino acid residues within the peptide sequences of distinct families have also been investigated. For example, the occurrence of hydrophobic amino acids such as leucine, glycine, valine, and isoleucine (L/G/V/I), which are more prevalent in the β-defensins family than in others, correlated with the formation and maintenance of their characteristic defensin motif and the consequent broad-spectrum and salt-stable antimicrobial activity [65]. Similarly, an elevated distribution of lysine (Lys) has been observed in peptides belonging to the Kunitz-type family, which has been associated with their capacity to bind with the Kv1.1 channel [66,67,68].

The 3D structure complements these findings by providing a framework for understanding how motifs within these putative peptides might fold into biologically active conformations. For example, the 3D structural model of Kunitz-type peptides is relatively stable, highlighting the conserved cysteine pattern and providing support for their ability to inhibit serine proteases and the Kv1.2 channel [69,70]. Similarly, the β-hairpin structure appeared as a frequent feature, hinting at the evolutionary conservation of these molecules, both within and outside the β-defensins [71,72]. The 3D structure of the putative peptides belonging to the eight major families demonstrates a certain level of conservation. Specifically, the ShK domain is characterized by a helix-loop-helix motif. In contrast, both β-defensins and BBH exhibit a conserved core structure, primarily composed of β-folds. The EGF peptides stand out due to their distinctive double-stranded β-sheet architecture, where β-sheets serve as the fundamental structural backbone. Furthermore, the α-β-β motif consistently appears within the 3D architecture of the putative peptides from the Kunitz-type, Kazal-like, ICK, and waprin families, creating a stable hydrophobic core typically structured by an α-helix positioned between two β-strands that may be either parallel or antiparallel. Upon aligning the models of all the putative peptides, we found that the fragments of proteins or peptides with the most similar 3D structures to those of the putative peptides predominantly originate from various types of sea anemones, with a smaller proportion derived from marine organisms such as sea cucumbers and sponges. Notably, there are some exceptional cases, such as the optimal putative peptide MD-312 from the β-defensins family, whose corresponding peptide is derived from terrestrial chickens, highlighting the diversity of the distribution of β-defensins in terrestrial and marine organisms [37,38,39]. In addition, all putative peptides and their corresponding peptides or protein fragments exhibit consistent cysteine patterns, indicating that cysteine indeed plays a very important role in maintaining the 3D structure of proteins [21,31,37,46,51,56,57,58,59,60,61,62]. The β-strands, through hydrogen bonding, create a fixed stratified structure, while interactions between side chains of the α-helix and β-sheet (involving hydrogen bonds, electrostatic forces, and hydrophobic interactions) contribute to enhanced stability in the peptide architecture and also play a role in its functionality. For instance, the α-helix may be involved in substrate recognition or the formation of catalytic sites, and this particular structure is found in numerous enzymes with catalytic activity [73]. The Kunitz-type and Kazal-like peptides, which act as serine protease inhibitors, share a highly conserved 3D structure (an α-helix and a multi-stranded anti-parallel β-sheet, with a stable central hydrophobic core) [18,19].

Through detailed molecular docking simulations, we assess the binding strength of the quaternary structure and analyze putative peptides that surpass known peptides in predicting activity. The ShK domain peptides primarily inhibit the Kv channels, with ShK and BgK serving as representative peptides of their family, both exhibiting inhibitory effects on the Kv1.3 channel [22,25]. Molecular docking simulations have differentiated between peptides within the ShK domain. MD-384 and MD-374 demonstrate a high affinity for the Kv1.3 channel and can form relatively stable peptide–protein complexes. Notably, MD-374 is predicted to form 27 favorable non-covalent bonds, including 18 hydrogen bonds, which are indicators of its potent inhibitory capability on the Kv 1.3 channel. Pharmaceuticals used to treat autoimmune disorders often cause systemic immunosuppressive effects, increasing the risk of opportunistic infections and neoplastic progression. By targeting the Kv1.3 channel, ShK and its derivatives specifically suppress CCR7-TEM lymphocytes, providing a strategy to mitigate widespread immunosuppression [23,74]. Molecular docking simulations indicate that MD-374 exhibits a potentially superior binding affinity for the Kv1.3 channel compared to that of ShK and BgK. Consequently, MD-374 may exhibit stronger inhibitory effects on the Kv1.3 channel, positioning it as a more promising lead compound for the treatment of autoimmune diseases than either ShK or BgK. Kunitz-type peptides, originating from the alkaline protease inhibitor family, have been identified across various taxonomic landscapes, indicating a ubiquitous biological role [75]. The bifunctional nature of Kunitz-type peptides, which involves serine protease inhibition and voltage-gated ion channel antagonism, represents a multifaceted mechanism potentially employed for predation and defense [76,77]. The complex formed by MD-364 and serine protease is the most stable, predicted to establish 16 favorable non-covalent bonds, including 10 hydrogen bonds. This finding is consistent with the known peptides of the Kunitz-type family evaluated in this study (Figure 7). Furthermore, MD-364 is anticipated to exhibit some binding affinity for the Kv1.2 channel, which is associated with pain [34]. Consequently, MD-364 emerges as a promising lead compound for the development of multifaceted drugs, focusing on serine protease inhibition and pain alleviation. β-defensins peptides play a crucial role in immune responses, exhibiting broad-spectrum antimicrobial and immunomodulatory activities [78,79]. MD-312 has been identified as a peptide belonging to the β-defensins family. It is speculated that MD-312 can form relatively stable protein–peptide complexes with the Kv1.2 or Nav1.7 channels, which may exert an inhibitory effect on both channels (Figure 9). Both the Kv1.2 and Nav1.7 channels are related to the process of pain production; consequently, MD-312 may have a strong analgesic effect. ICK peptides, characterized by four pairs of disulfide bonds, play a key role in modulating the Nav and Cav channels [51,52,53]. MD-428 and MD-429 have been identified as peptides belonging to this family. Based on the predicted docking results, these putative peptides can form stable complexes with the Nav1.7 channel (Figure 11), potentially exerting a strong inhibitory effect on the Nav1.7 channel. This could provide valuable insights for the development of analgesics, as the Nav1.7 channel is associated with the pain-production process. The advantage of these biologically derived peptide substances is that they can potentially avoid many of the side effects commonly seen with clinically used analgesics by inhibiting the ion channels [80]. Molecular docking simulation serves as a key bridge between structural prediction and practical biological significance [81], with molecular docking technology used to compare putative peptides within the family and highlight top candidate peptides from different families.

## 4. Materials and Methods

### 4.1. Specimen Collection

These sea anemones were collected from the Paracel Islands (7.8 km^2^), located at [15°46′ N, 111°11′ E] in the South China Sea. A total of three sea anemones were collected and identified as *M. doreensis*. The sea anemone *M. doreensis* has been identified based on its external morphological features. It closely resembles the sea anemone *Cribinopsis crassa*, but the tentacles of *Cribinopsis crassa* are more numerous and stiffer. Its mouthparts are usually visible, and the adult diameter can usually reach up to 50 cm. Its tentacles are spaced farther apart than are those of similar sea anemones. They present in various colors, but the base is usually orange to red. The specimens were cultivated in an indoor water tank for at least three weeks, and then tweezers and a surgical knife were used to cut the tentacles and columnar tissues of three individuals, which were then mixed separately before being stored in liquid nitrogen.

### 4.2. Transcriptome Construction and Quality Checking

Total RNA was extracted using the RNeasy Mini Kit (Qiagen, Duesseldorf, North Rhine Westphalia, Germany). The Illumina sequencing library was constructed using the NEBNext^®^ Ultra™ RNA Library Prep Kit for Illumina^®^ (NEB, Beverly, MA, USA), and the qualified libraries were sequenced using the Illumina Novaseq6000 (Illumina, San Diego, CA, USA) high-throughput sequencing platform. The sequencing strategy was PE150 (Pair End 150), and the sequencing data volume for each sample was not less than 6 Gb [82,83].

### 4.3. Gene Annotation

The prediction of the coding region of the unigene uses three types of forward and reverse reading frames, which may generate a total of six types of encoded protein sequences to be predicted. After obtaining the encoded protein sequences, they were compared with the Nr (non-redundant protein sequences database) and the UniProt protein database. The encoding method of the gene with the best comparison (maximum comparison score) is selected. To identify potential toxin transcripts, the translated transcriptome data are cross-aligned with the assumed animal toxin sequences, retrieving information from the five major public databases (Nr, UniProt, KEGG, GO, and KOG/COG) [84,85,86].

### 4.4. Classification of Proteins and Peptides of M. doreensis

To identify the protein family (domain) in *M. doreensis*, we first retrieved the *M. doreensis* genome sequences, including 287,880 protein-coding genes from the Pfam database (https://pfam-legacy.xfam.org/, accessed on 8 October 2024) using local BLASTP (with an e-value of 1 × 10^−10^, -max_target_seqs 2). The related protein-coding genes from *M. doreensis* were divided by SignalP 5.0 (https://services.healthtech.dtu.dk/services/SignalP-5.0/, accessed on 1 September 2024) as signal and mature regions. The putative proteins and peptides must be sequences containing predicted signal regions and mature regions. A total of 4239 protein-coding genes were collected and then aligned using MUSCLE [87] to build a hidden den markov model (HMM) profile using hmmbuild in HMMER v3.0 (http://hmmer.janelia.org/static/binaries/hmmer3.0_windows.zip, accessed on 4 October 2024). The complete proteins family (domain) in *M. doreensis* was identified using hmmsearch program (with an e-value of 1 × 10^−3^) by searching the full protein-coding gene sequences of the *M. doreensis* genome, based on the HMM profile. The candidate proteins were further confirmed using Pfam [88] programs, combining them with their corresponding cysteine frameworks to predict the family of sea anemone proteins and peptides. The redundant proteins were manually removed (Table S2). In our current research, peptides with less than 120 amino acids are defined as peptides.

### 4.5. Phylogenetic Analyses

The mature regions of 40 putative peptides were aligned using MEGA 7.0.14. A phylogenetic tree was established using the Maximum Likelihood (Bootstrap Method 2000).

### 4.6. Alignment

Multiple sequence alignment was performed using Snapgene Viewer (aligned using MUSCLE [87], with the results marked with color highlighting based on the physicochemical properties and the consensus, with a threshold of >50%), and the sequence was exported in the FASTA format [89]. The sequence homology (similarity and identity) was analyzed using the manual calculation method.

### 4.7. AlphaFold2 Modeling and Model Alignment

We logged in to the AlphaFold Colab website and entered the sequence of the estimated protein to obtain its model [90] (https://colab.research.google.com/github/sokrypton/ColabFold/blob/main/AlphaFold2.ipynb, accessed on 6 October 2024). Using the Foldseek tool for model alignment, we accessed the Foldseek website [91] (https://search.foldseek.com/search, accessed on 8 October 2024), uploaded the model to be aligned (in PDB or CIF format), opened the Databases & Search settings, selected eight databases (AlphaFold/Proteome, AlphaFold/Swiss-Prot, AlphaFold/UniProt50, BFMD, CATH50, GMGCL, MGnify-ESM30, PDB100), selected the 3Di/AA mode, and clicked “search” to get the results.

### 4.8. Molecular Docking and Eyelash Diagram

The interaction between the key targets and the main active components was further investigated using molecular docking. Energy minimization of the 3D structures was performed using ChemBio 3D. The crystal structures of the receptors were downloaded from the RCSB Protein Data Bank (http://www.pdb.org/, accessed on 8 October 2024) and subsequently modified using Discovery Studio 2019. The modifications included hydrogen addition, ligand and water removal, amino acid optimization, and charge computation. Discovery Studio 2019 was employed for the docking and visualization of the docking results. The required functional modules include Dock Proteins with ZDOCK (rigid protein docking algorithm) and Refinement with RDOCK (evaluating the binding configuration of the complex), and the data files are the receptor proteins and peptides. The ZDOCK parameter is set to Angular Step Size 15, with a sample number of 3600 binding configurations (when the sampling angle is selected as 6 degrees, there are 54,000 samples, resulting in more accurate prediction results, but requiring more time). In addition, for such a small binding configuration, for improved results, the RMSD Cutoff should be set to 6.0, the Interface Cutoff to 9.0, and the Maximum Number of Clusters to 60. The Zrank parameter should be set to False, while the other parameters will remain at their default values. Select the pose with the highest score from the 10 largest clusters in the ZDOCK results required by the RDOCK (default parameters). The smaller the RDOCK score, the closer it is to the true docking conformation. The results were analyzed using LigPlot+ to evaluate the accurate binding interactions between the ligands and the receptors. All the targets and their PDB IDs used in the modeling are included in Table S3.

## 5. Conclusions

This study reports, for the first time, a comprehensive transcriptomic analysis of different tissues of *Macrodactyla doreensis*. A total of 4239 transcripts were identified, and a database of putative proteins (81.53%) and peptides (18.47%) was preliminarily constructed. Immunoglobulin, mainly distributed in the column, comprised the greatest content of the putative protein, along with putative peptides containing the ShK domain, which were mainly distributed in the tentacle. Bioinformatics was further integrated to screen for putative peptides with potentially high activity. The optimal peptides selected from this database are MD-374, MD-364, MD-312, and MD-429, which belong to the ShK domain, Kunitz-type, β-defensins, and ICK families, respectively. It is through such research that we can bridge the gap between the sequence, structure, and target, providing direction for further in-depth research on the peptides of *Macrodactyla doreensis*.

## Data Availability

The transcriptome data of *M. doreensis* have been uploaded to the National Center for Biotechnology Information (NCBI) repository (BioProject: PRJNA1134430; SRA accession: SRR29774726 and SRR29774727).

## Supplementary Materials

The following supporting information can be downloaded at: https://www.mdpi.com/article/10.3390/md22100470/s1, Table S1. Sequencing statistics and assembly summary of *M. doreensis* transcriptome; Table S2. Classification of proteins and peptides of *M. doreensis*; Table S3. The targets and their PDB IDs used in docking; Table S4. The protein or peptide exhibiting the most structurally similar fragment to each of the putative peptides; Figure S1. The number of gene annotations between different databases (GO; KEGG; Nr; KOG) for the *M. doreensis* transcriptome; Figure S2. Deduced BBH transcripts from *M. doreensis*; Figure S3. Molecular docking analyses revealed the binding interaction between BBH peptides and the Kv1.1 channel; Figure S4. Deduced Kazal-like transcripts from *M. doreensis*; Figure S5. Molecular docking analyses revealed the binding interaction between Kazal-like peptides and the serine proteinase receptor; Figure S6. Deduced EGF transcripts from *M. doreensis*; Figure S7. Molecular docking analyses revealed the binding interaction between EGF peptides and EGFR; Figure S8. Deduced waprin transcripts from *M. doreensis*; Figure S9. Phylogenetic analysis of the putative ShK domain, Kunitz-type, and β-defensins peptides derived from four common sea anemones in the South China Sea.
