# Supplementary material for: Revealing the Diversity of Sequences, Structures, and Targets of Peptides from South China Sea Macrodactyla doreensis Based on Transcriptomics
Source: Mar Drugs. 2024 Oct 12;22(10):470. doi: 10.3390/md22100470 (PMC11509556; doi:10.3390/md22100470)
Supplement: Supplementary file 1 [file marinedrugs-22-00470-s001.zip › Figure S1-9.pdf]

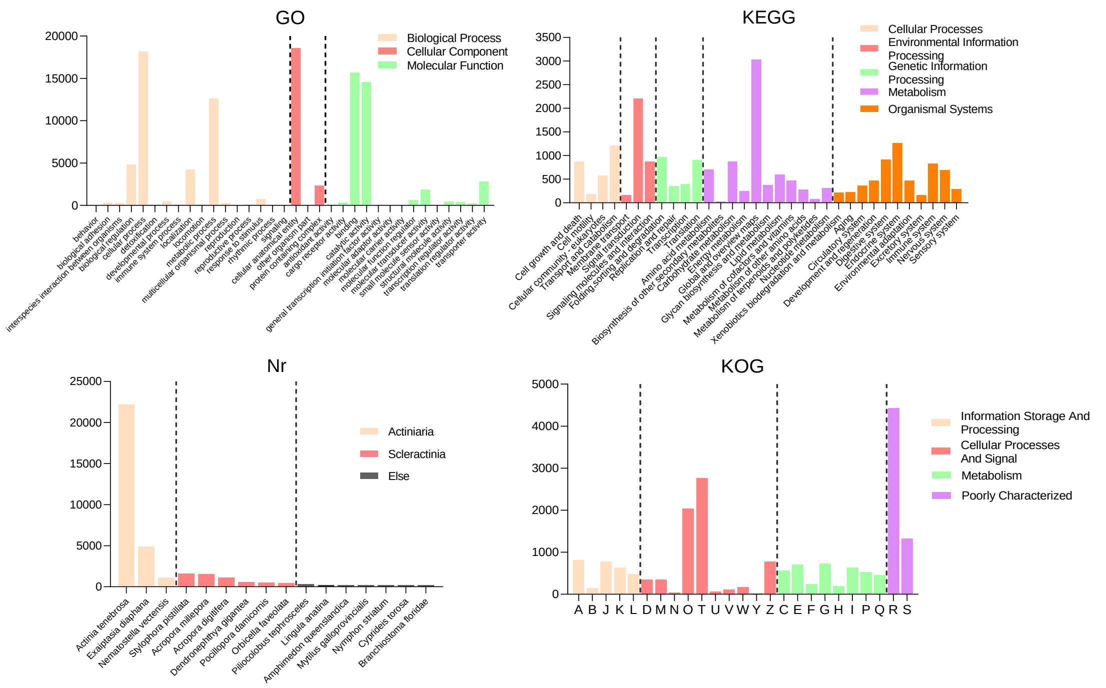

Figure S1. The number of gene annotations between different databases (GO; KEGG; Nr; KOG) for the *M. doreensis* transcriptome.

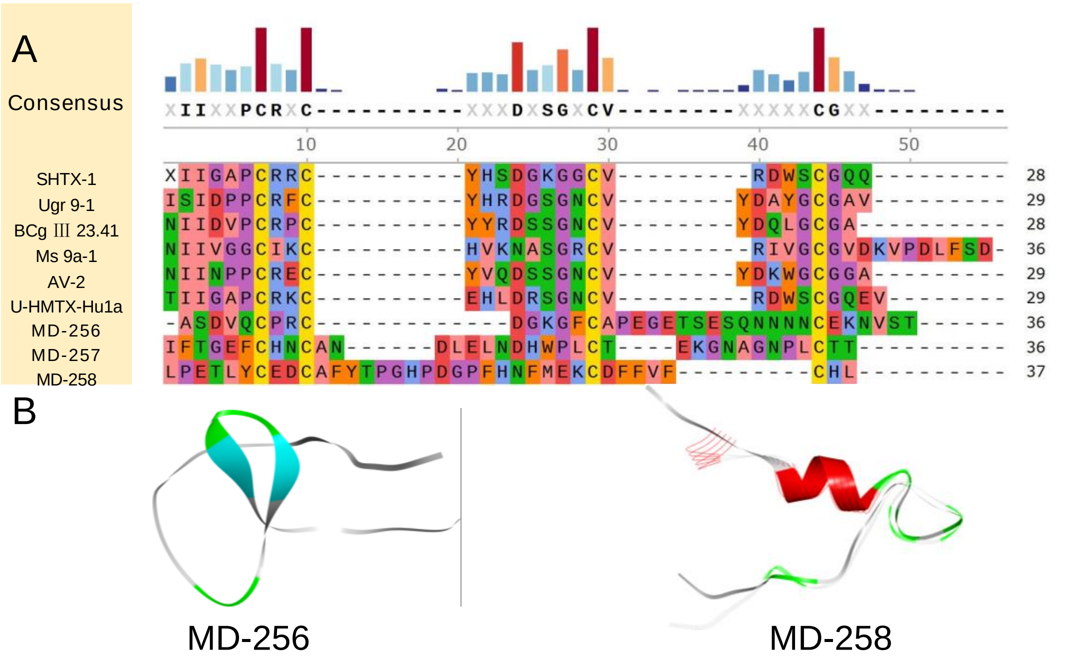

Figure S2. Deduced BBH transcripts from *M. doreensis*. (A) Multiple sequence alignment of known BBH peptides and corresponding putative mature peptidess. The representative BBH peptides shown are SHTX-I (Uniport: P0C7W7) from the Saddle carpet anemone or Haddon's sea anemone *Stichodactyla haddoni*, Ugr 9a-1 (Uniport: S4S1V7; PDB: 2LZO) from the Painted anemone *Urticina grebelnyi*, BCgIII23.41 (Uniport: P86467; AlphaFold: AF-P86467-F1) from the Sea anemone *Bunodosoma cangicum*, Ms 9a-1 (Uniport: A0A1R3S3A8; AlphaFold: AF-A0A1R3S3A8-F1) from the Brown sea anemone or Frilled sea anemone *Metridium senile*, AV-2 (Uniport: P0DMZ9; AlphaFold: AF-P0DMZ9-F1) from the Snakelocks anemone *Anemonia viridis*, U-HMTX-Hdu1a (Uniport: C0HJB4; AlphaFold: AF-C0HJB4-F1) from the Sea anemone or Stichodactyla duerdeni *Homostichanthus duerdeni*. (B) The putative peptide models (flat representation) of BBH predicted by Alphafold2 (the line model that overlaps with them is the best peptide obtained in the model alignment).

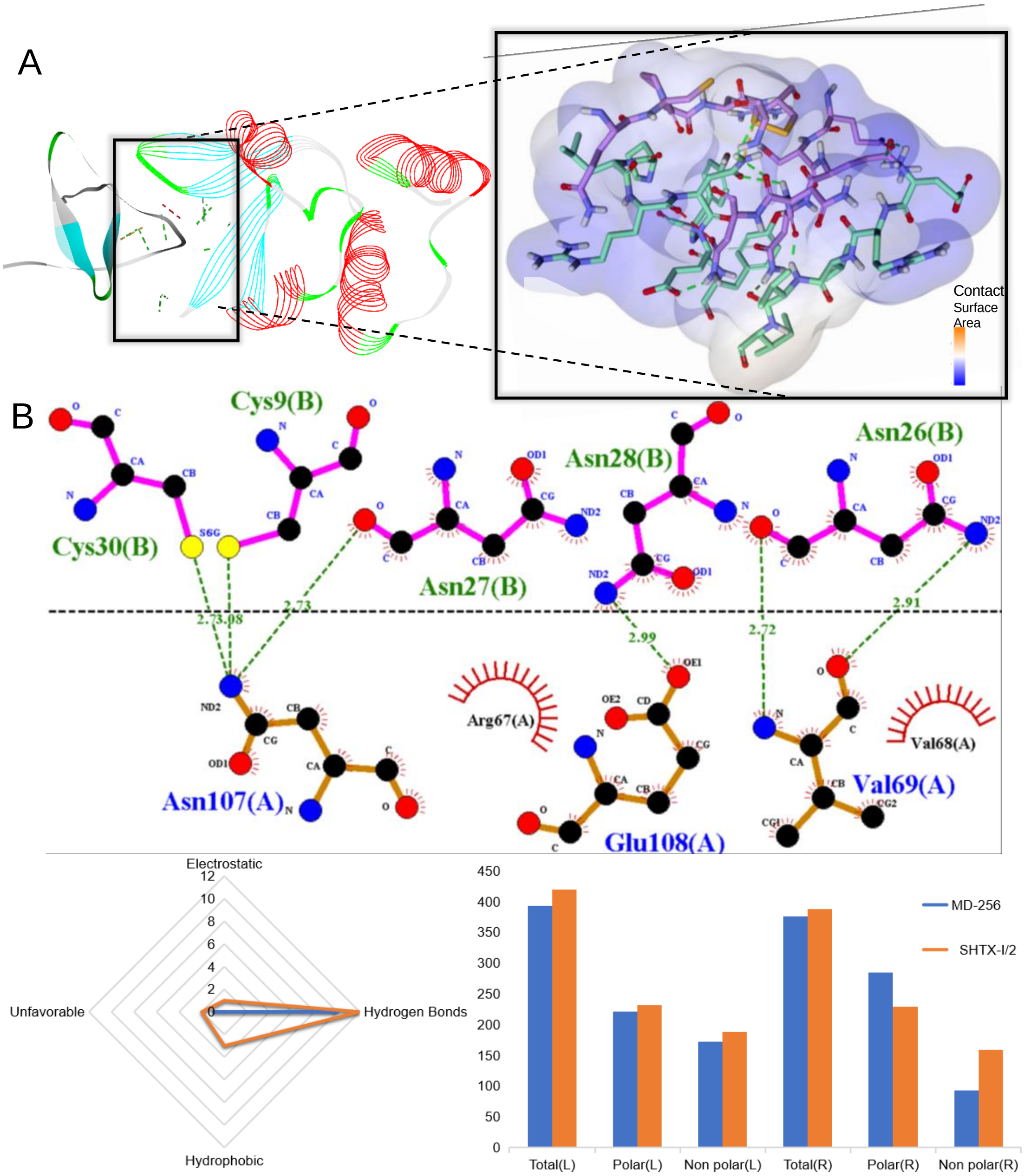

Figure S3. Molecular docking analyses revealed the binding interaction between BBH peptides and Kv1.1 channel. (A) A docking pattern diagram illustrating MD-256 (flat representation) and Kv1.1 channel (PDB: 1A68) (line representation), while a more comprehensive three-dimensional diagram displaying the docking locations and contact surface area of MD-256 (colored purple) and Kv1.1 channel (colored green). (B) An eyelash (2D) diagram depicting the key docking sites of MD-256 (colored blue) and Kv1.1 channel (colored green). (C) A radar chart illustrating the number of non-bond interactions between BBH peptides and Kv1.1 channel, while a 3D surface plot depicting the contact surface area. Ligand (L); Receptor (R).

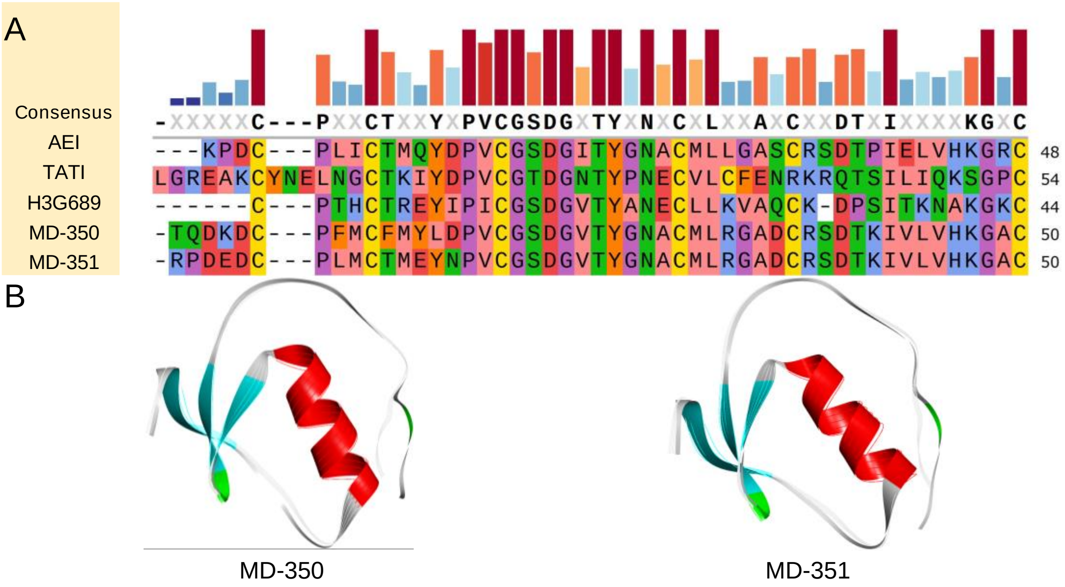

Figure S4. Deduced Kazal-like transcripts from *Macrodactyla doreensis*. (A) Multiple sequence alignment of Kazal-like peptides and corresponding putative mature peptides. The representative Kazal-like peptides shown are AEI (Uniport: P16895; PDB: 1Y1B) from the Mediterranean snakelocks sea anemone *Anemonia sulcata*, TATI (Uniport: P00995; PDB: 1HPT) from the Human *Homo sapiens*, H3G689 (Uniport: H3G689; AlphaFold: AF-H3G689-F1) from the *S*udden oak death agent *Phytophthora ramorum*. (B) The putative peptide models (flat representation) of Kazal-like predicted by Alphafold2 (the line model that overlaps with them is the best peptide obtained in the model alignment).

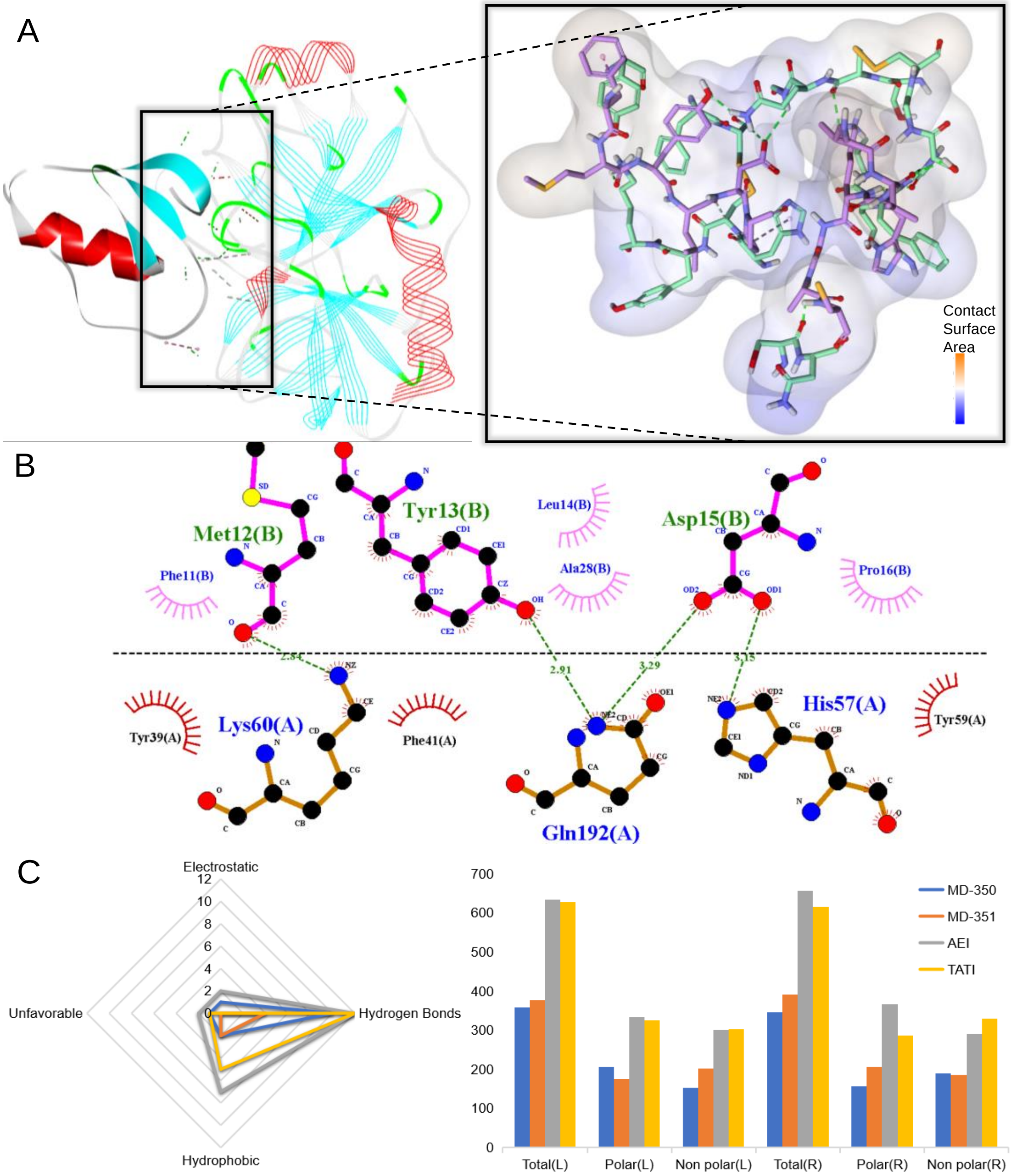

Figure S5. Molecular docking analyses revealed the binding interaction between Kazal-like peptides and serine proteinase receptor. (A) A docking pattern diagram illustrating MD-350 (flat representation) and serine proteinase receptor (PDB: 5PTP) (line representation), while a more comprehensive three-dimensional diagram displaying the docking locations and contact surface area of MD-350 (colored purple) and serine proteinase receptor (colored green). (B) An eyelash (2D) diagram depicting the key docking sites of MD-350 (colored blue) and serine proteinase receptor (colored green). (C) A radar chart illustrating the number of non-bond interactions between Kazal-like peptides and serine proteinase receptor, while a 3D surface plot depicting the contact surface area. Ligand (L); Receptor (R).

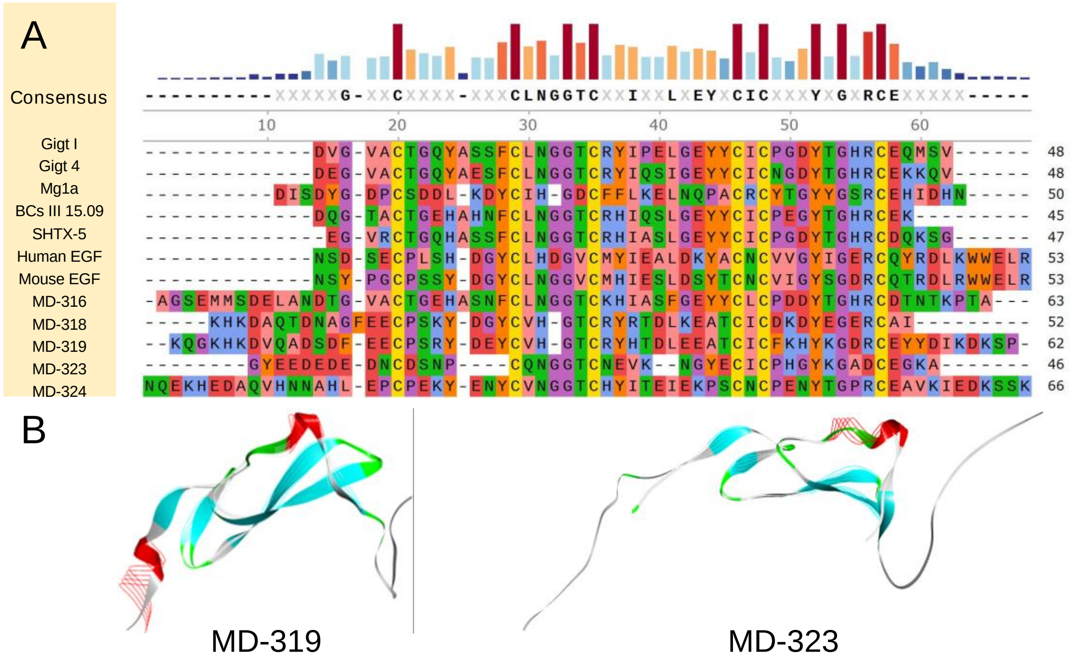

Figure S6. Deduced EGF transcripts from *Macrodactyla doreensis*. (A) Multiple sequence alignment of known EGF peptides and corresponding putative mature peptidess. The representative EGF sequences shown are human EGF (Uniport: P01133; PDB: 1P9J) from the Human *Homo sapiens*, mouse EGF (Uniport: A0A0G2JDT8; PDB: 1EPG) from the Mouse *Mus musculus*, Gigt1 (Uniport: Q76CA1; AlphaFold: AF-Q76CA1-F1) from the Giant carpet anemone *Stichodactyla gigantea*, Gigt4 (Uniport: P0DMY9; AlphaFold: AF-P0DMY9-F1) from the Snakelocks anemone *Anemonia viridis*, BCs III 15.09 (Uniport: P86468; AlphaFold: AF-P86468-F1) from the Sea anemone *Bunodosoma caissarum*, SHTX-5 (Uniport: B1B5J0; AlphaFold: AF-B1B5J0-F1) from the Haddon's sea anemone or Saddle carpet anemone *Stichodactyla haddoni*. (B) The putative peptide models (flat representation) of EGF predicted by Alphafold2 (the line model that overlaps with them is the best peptide obtained in the model alignment).

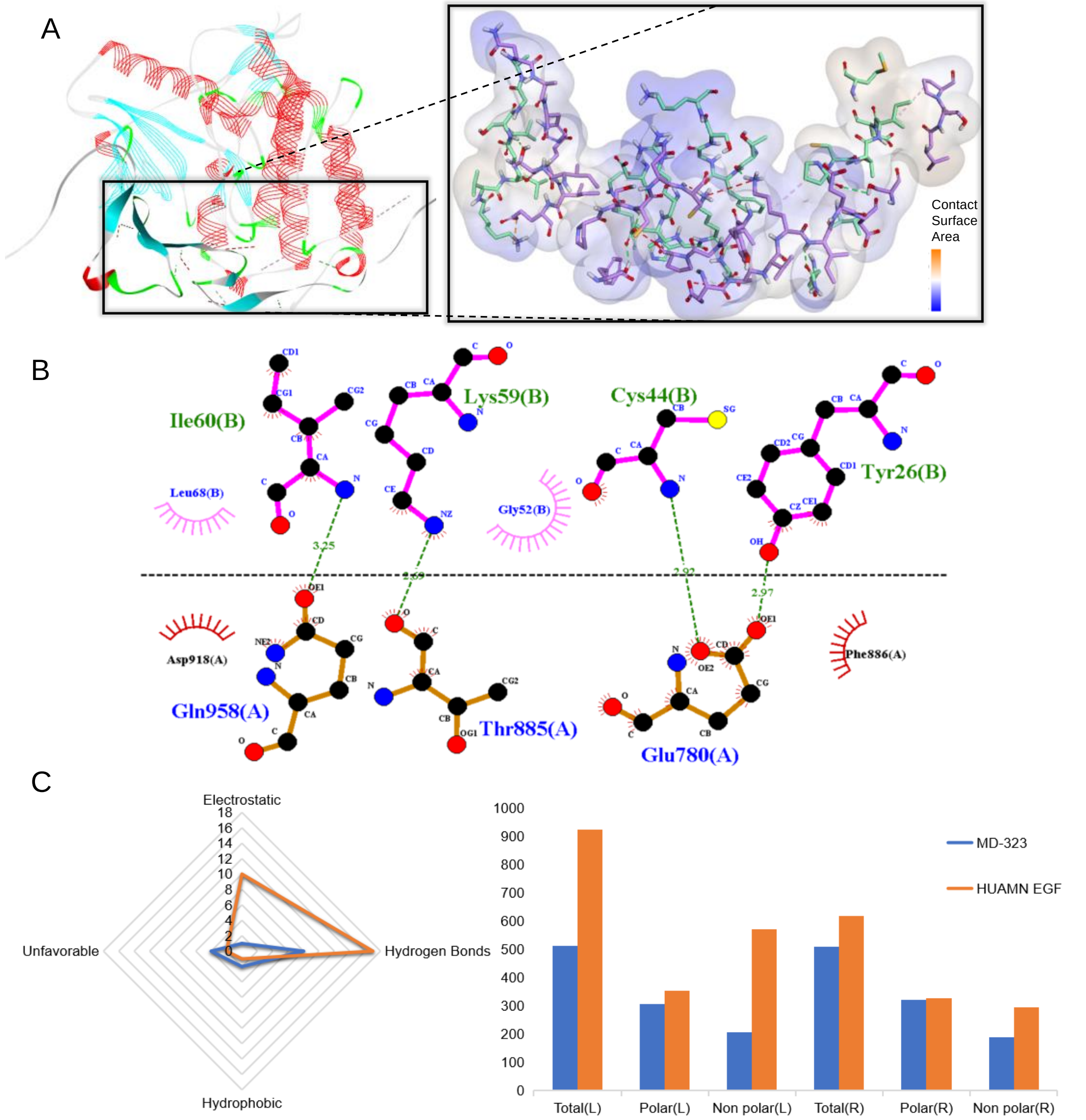

Figure S7. Molecular docking analyses revealed the binding interaction between EGF peptides and EGFR. (A) A docking pattern diagram illustrating MD-323 (flat representation) and EGFR (PDB: 1M14) (line representation), while a more comprehensive three-dimensional diagram displaying the docking locations and contact surface area of MD-323 (colored purple) and EGFR (colored green). (B) An eyelash (2D) diagram depicting the key docking sites of MD-323 (colored blue) and EGFR (colored green). (C) A radar chart illustrating the number of non-bond interactions between EGF peptides and EGFR, while a 3D surface plot depicting the contact surface area. Ligand (L); Receptor (R).

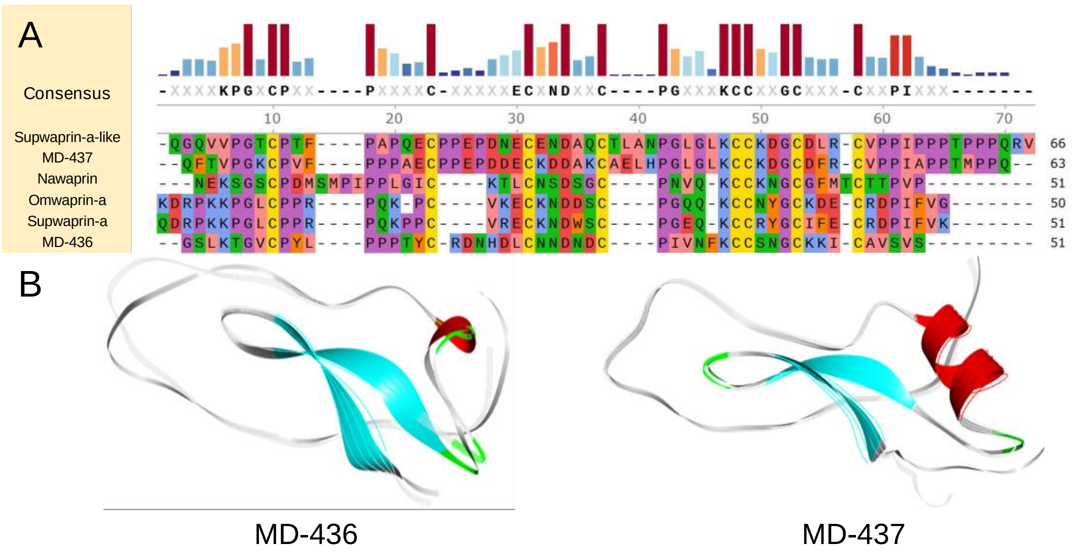

Figure S8. Deduced waprin transcripts from *Macrodactyla doreensis*. (A) Multiple sequence alignment of known waprin peptides and corresponding putative mature peptides. The representative Waprin peptides shown are Supwaprin-a-like from the starlet sea anemone *Nematostella vectensis*, Nawaprin (Uniport: P60589; PDB: 1udkA) from the Black-necked spitting cobra *Naja nigricollis*, supwaprin-a (Uniport: B5KGY9; AlphaFold: AlphaFold: AF-P0DMY9-F1) from the Lowland copperhead snake or Hoplocephalus superbus *Austrelaps superbus*, Omwaprin-a (Uniport: P83952; PDB: 3nggB) from the Inland taipan or Diemenia microlepidota *Oxyuranus microlepidotus*. (B) The putative peptide models (flat representation) of waprin predicted by Alphafold2 (the line model that overlaps with them is the best peptide obtained in the model alignment).

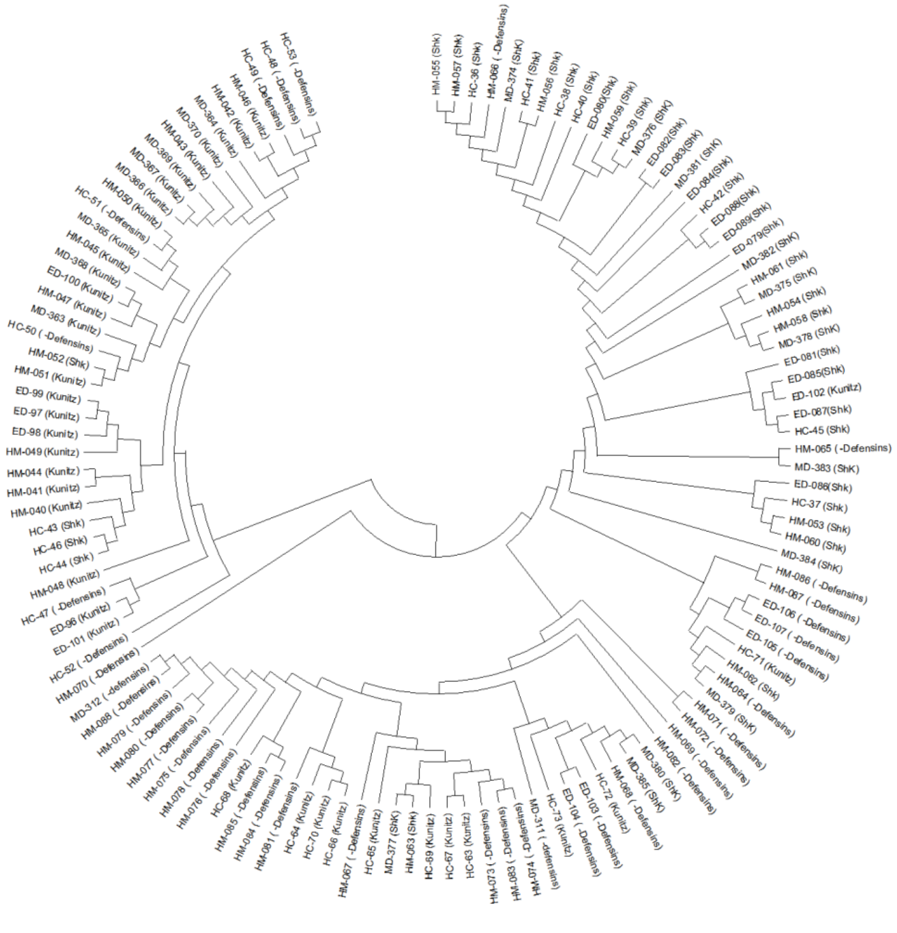

Figure S9. Phylogenetic analysis of putative ShK domain, Kunitz-type, and β-Defensins peptides derived from four common South China Sea anemones.
